# Evaluating the effect of the plan of national syphilis control in controlling the syphilis epidemic in Jiangsu, China 2010–2020

**DOI:** 10.3389/fpubh.2023.1281229

**Published:** 2023-12-21

**Authors:** Lingen Shi, Liping Chen, Xiaoyan Liu, Haiyang Hu, Yuheng Chen, Yunting Chen, Zhi Zhang, Ying Zhou, Jing Lu, Dandan Yang, Wenhui Guan

**Affiliations:** ^1^Jiangsu Provincial Center for Disease Control and Prevention, Institute for STI and HIV Control and Prevention, Jiangsu, China; ^2^Medical School, Nanjing University, Jiangsu, China

**Keywords:** early syphilis, congenital syphilis, trends, evaluating, epidemic

## Abstract

**Background:**

Starting in 2010, the Chinese government initiated a 10-year syphilis control plan, called the national syphilis control plan (NSCP), to address the emerging threat of syphilis. We aimed to evaluate the effect of the NSCP plan on syphilis control in Jiangsu, China.

**Methods:**

The temporal trends of syphilis incidence, prevalence and rate of condom use were estimated by Joinpoint regression with average annual percent change (APC) and average annual percentage (APPC). A Chi-square test was conducted to analyze the outcomes in different subgroups. ArcGIS was used to analyze the spatiotemporal distribution of syphilis incidence.

**Results:**

Geographically, early and congenital syphilis incidence decreased significantly in all areas of the province during the period of NSCP. Early syphilis incidence decreased from 21.1 to 8.8 (APC: −7.5, 95%CI: −8.6, −6.5, *p* < 0.001) per 100,000 people, and congenital syphilis decreased from 63.6 to 4.1 (APC: −14.8, 95%CI: −20.8, −8.4, *p* < 0.01) per 100,000 newborns from 2010 to 2020. Also, syphilis prevalence reduced from 13.4 to 3.8% (APC: −8.7, 95%CI: −12.1, −5.0, *p* = 0.001) among men who have sex with men, from 5.3 to 1.7% (APC: −7.9, 95%CI: −11.7, −3.8, *p* = 0.002) among female sex workers and remained under 1.0% with slight variations among pregnant women (APC: 0.3, 95%CI: −4.3, 5.1, *p* = 0.877) from 2010 to 2020. 0.2% (2,436) of pregnant women who received free syphilis testing during pregnancy were diagnosed with current syphilis infection, and 97.0% (2,555) of newborns in the province were delivered to women diagnosed with syphilis. 91.8% (2,346) of live babies and about 90% of diagnosed patients received complete standard syphilis diagnosis and treatment services.

**Conclusion:**

Trends of early syphilis incidence and syphilis prevalence show a considerable decreasing trend among almost all the key populations after implementing NSCP. Congenital syphilis has significantly decreased as well and hence, the NSCP program should be sustained and strengthened to control the syphilis epidemic in China further.

## Introduction

In China, after nearly eliminating syphilis in the 1960s, syphilis has seen a resurgence since the early 1980s, with a dramatic increase since 1990s (1). Estimates of syphilis prevalence showed a 3.3 times increased to 327,433 (24.66 cases per 100,000 residents) since 2004, becoming one of the top three reported infectious diseases in China (2, 3). Currently, syphilis has emerged as a major public health problem among high-risk populations in China (4), with a reported prevalence of 14.6% among men who have sex with men (MSM), 12.5% among female sex workers (FSW), and 6.8% among drug users (DU) in 2006 (4) and 5.3% among commercial sex male clients (CSMC) in 2010 (5). Similarly, a high syphilis prevalence of 6.57, 18.09, and 18.72% was observed among DU, FSW and MSM in Jiangsu province from 1999 to 2003, respectively (6, 7). This alarming rise in the epidemic among key populations threatened the general population (8, 9).

To curb the emerging epidemic, the Chinese government initiated a 10-year national syphilis control plan in 2010. This plan aimed to decrease early syphilis incidence (primary and secondary syphilis incidence), and eliminate congenital syphilis to less than 15/100,000 newborns nationally by the end of 2020. Comprehensive serial measures and actions to achieve this goal included (1) expanding the coverage of syphilis-related prevention knowledge and awareness among key populations and the general population, (2) screening all surgery patients, in-patients, antenatal care women (ANC) and high-risk groups (especially MSM, FSW) for syphilis, (3) encouraging Provider-initiated integrated HIV and syphilis testing and counseling (PITC) for patients with a history of high-risk sexual behaviors in STI clinics. Meanwhile, each county dedicated at least one medical institute that provided standard medical services, including screening, testing, and treatment, for syphilis patients to reduce the wait time of patients seeking syphilis-related care. In addition, interventions to promote partner notification and reduce syphilis-related stigma/discrimination among STI patients were implemented alongside syphilis awareness creation among the general population. The NSCP specified eight milestones for achievement by 2015 and 2020 (Supplementary Box S1).

This study aimed to assess the provincial-level implementation of syphilis control programming and achievement of the 2020 milestones in this national syphilis control plan.

## Materials and methods

### Data sources

**Figure 1:**
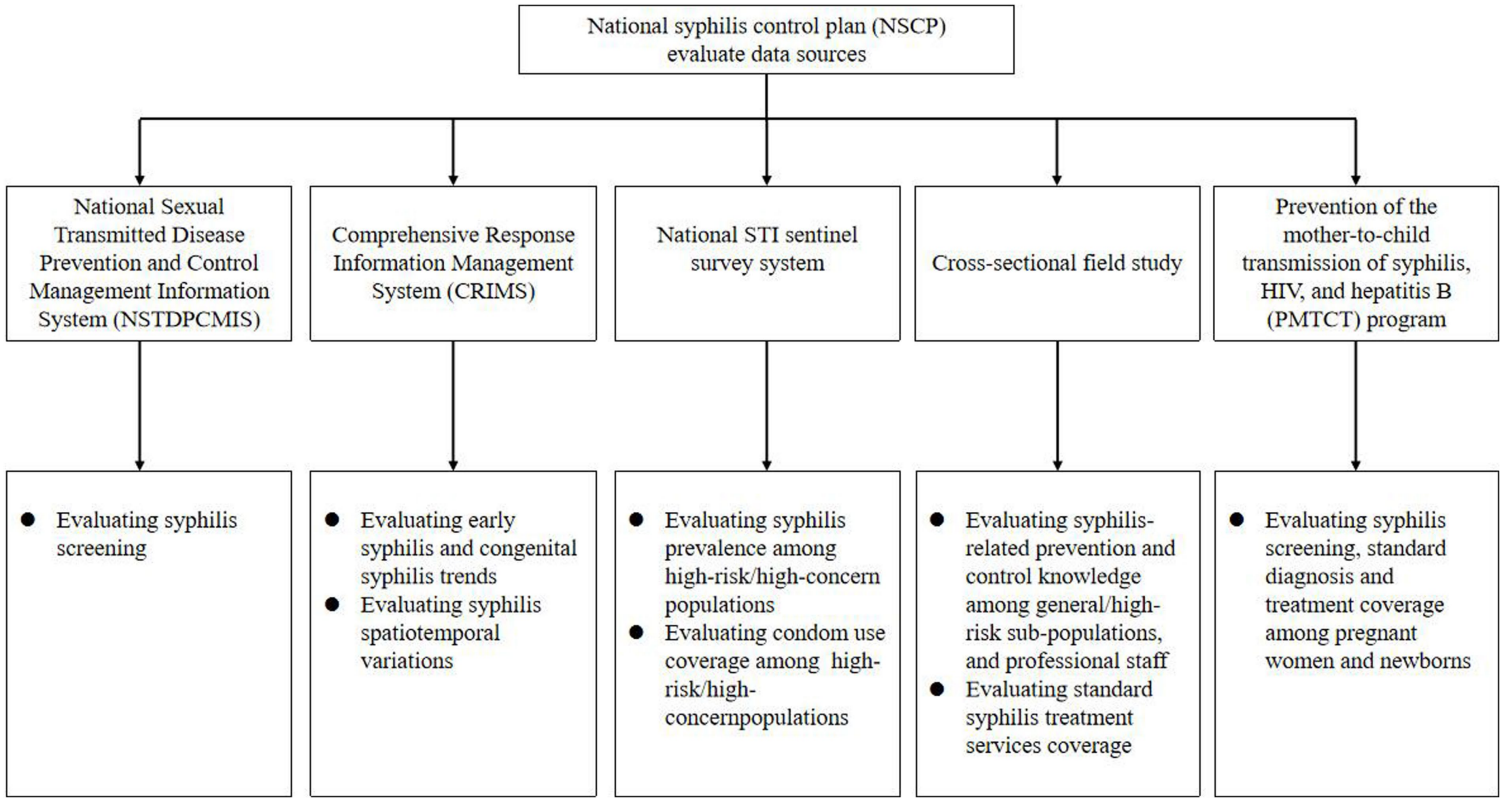
Flowchart of national syphilis control plan (NSCP) evaluating system in Jiangsu, China.

Multiple date sources were extracted from 13 cities of 95 counties or districts to evaluate the NSCP plan’s effect in Jiangsu, China (Figure 1).

Syphilis screening related data to assess the trend in syphilis screening was obtained from the National Sexual Transmitted Disease Prevention and Control Management Information System (NSTDPCMIS),. established to collect syphilis screening data quarterly in 2015. As of 2020, more than 2000 hospitals in Jiangsu province could provide STI clinical services, including counseling, screening, diagnosis, and treatment for syphilis. Furthermore, syphilis data from other health centers can also be accessed and collected by NSTDPCMIS. This study categorized screening test sites as clinical, key, and general sub-populations. Data from participants sought additional clinical services during the facility visit for syphilis testing (like tests for other STIs, surgery, and medical examinations provided to in-patients) were categorized as “clinical sub-population” data during analysis. Syphilis screening data from community sites (like voluntary counseling and testing “VCT” centers, Methadone maintenance treatment centers, voluntary blood donor centers, detention centers, and prison centers) were categorized as “Key population” data, and reports of syphilis screening obtained via other means (pre-marital examination centers, conscription physical examination centers, international travelers physical examination centers, occupational physical examination centers and others) were grouped as “general sub-population” data.

Syphilis-related data to evaluate the trend of incidence of syphilis and spatiotemporal variations trend from 2010 to 2020 was retrieved from the Comprehensive Response Information Management System (CRIMS), an online system developed by the Chinese government to collect 33 pre-specified infectious diseases in real-time since 2004. From any health facilities, syphilis has been one of the mandatory diseases for case-reporting in China. All the syphilis cases diagnosed by clinicians were reported via CRIMS within 24 h.

Data to evaluated the syphilis prevalence and condom use coverage among the high-risk sub-populations was obtained from the China national STI sentinel survey, which is conducted annually in the second quarter in Jiangsu province since 2008. The sentinel surveys aim to monitor the prevalence of STIs (including HIV, syphilis, Genital *Chlamydia trachomatis*, and *Neisseria gonorrhoeae*), sexual behaviors, and history of drug use and STI diagnosis among all high-risk populations (including FSW, MSM, DUS and CSMC) and high-concern populations’ (pregnant women (PRG), younger college students, migrant male people). There were 130 sentinel sites in the 13 cities, which enrolled more than 120,000 participants in the 2020 sentinel survey.

### Syphilis testing policy

**Figure 2:**
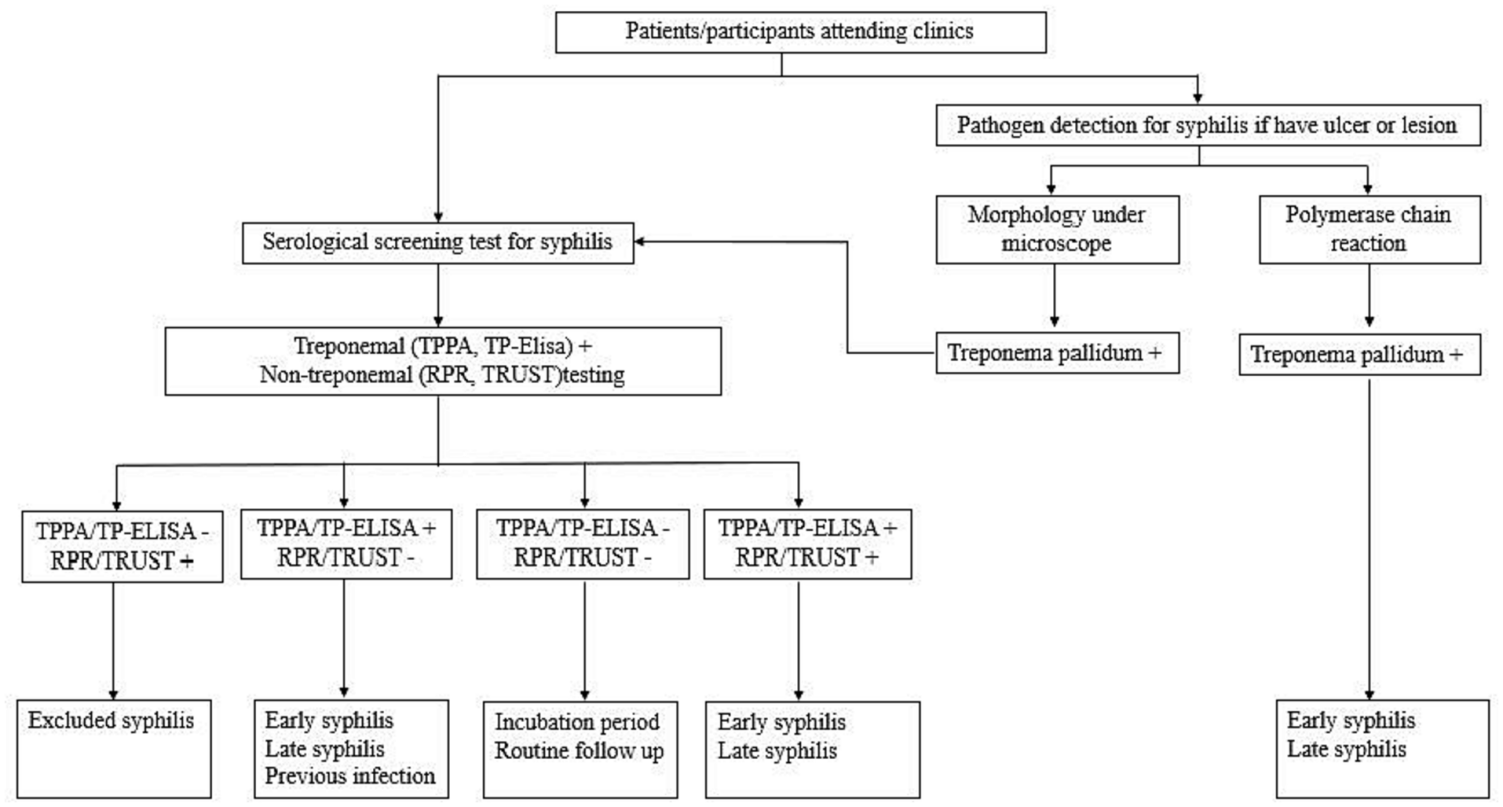
Flowchart of the syphilis test/diagnosis for the general population/ participants enrolled in the sentinel surveillance system in Jiangsu.

All participants in the sentinel surveillance system received free treponemal and non-treponemal syphilis testing at the same time syphilis serology testing. Treponemal tests were conducted using the *Treponema Pallidum* particle agglutination assay (TPPA) or the *Treponema Pallidum* Enzyme Linked Immunosorbent Assay (TP-ELISA). The non-treponemal tests used macroscopic rapid plasma reagin (RPR) or tolulized red unheated serum test (TRUST). Participants with positive serum antibody tests from any one of the tests were referred to the STI clinic for linkage-to-care (diagnosis, treatment, and follow-up). Patients could also get etiology detection services (including morphology or polymerase chain reaction) in hospitals or STI clinics (Figure 2).

**Figure 3:**
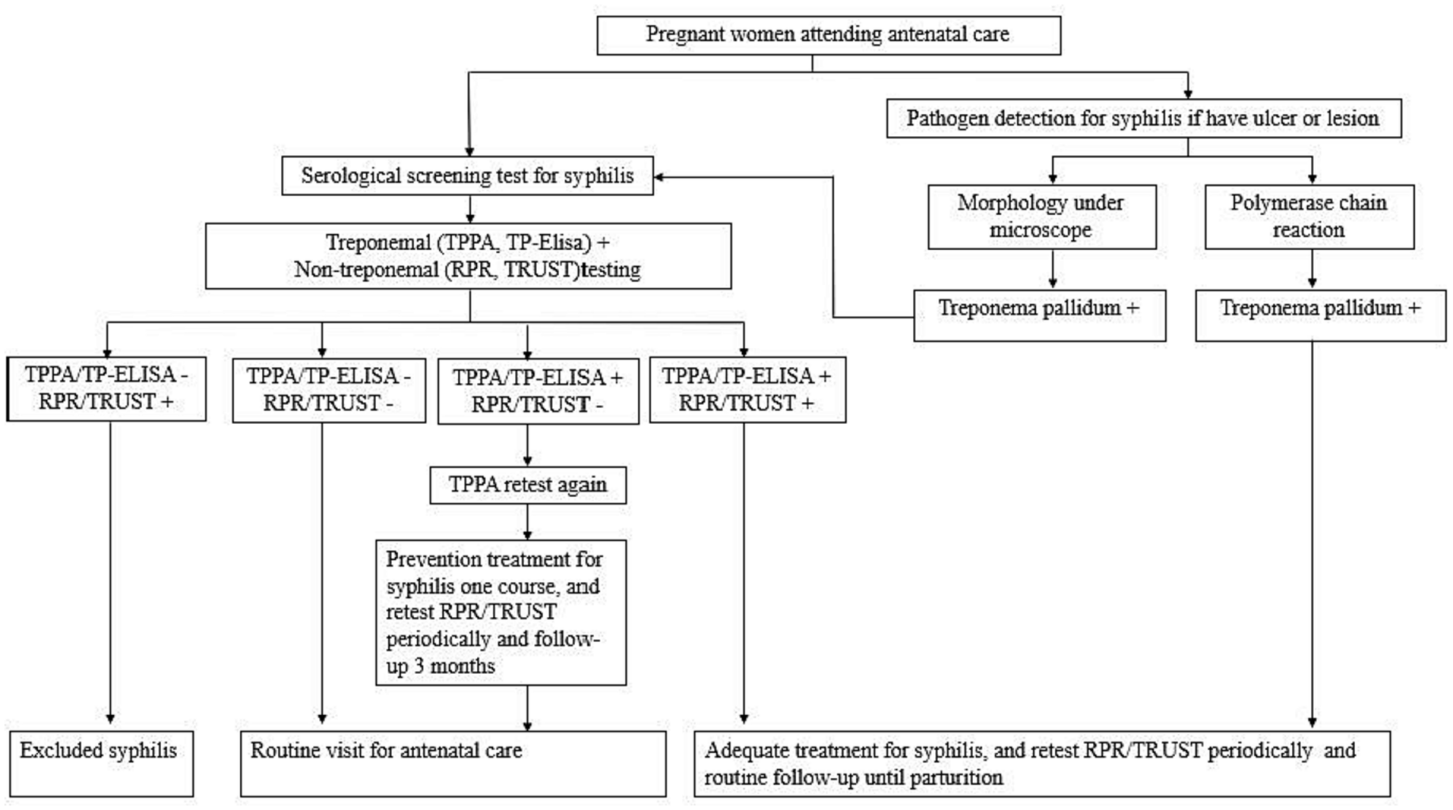
Flowchart showing the prevention of mother-to-child transmission (PMTCT) services for syphilis testing/diagnosis in Jiangsu.

All pregnant women seeking prenatal care were first registered into the prevention of the mother-to-child transmission of syphilis, HIV, and hepatitis B (PMTCT) program. In the PMTCT program, Syphilis screening, diagnosis in pregnant women, congenital syphilis cases, and infant follow-up among PMTCT registered women were tracked to evaluate the effects of NSCP. Pregnant women also received a treponemal and non-treponemal serological screening at the first prenatal care visit, and those diagnosed with maternal syphilis (both treponemal and non-treponemal are positive) received standard treatment (benzathine penicillin or other antibiotics). Pregnant women with single TPPA-positive result also received one-course benzathine penicillin (or other antibiotics) for preventive treatment (Figure 3).

### Diagnosis of syphilis

Trained clinicians assessed a patient’s syphilis infection risk through their sexual history and physical manifestation of symptoms and relied on laboratory results for confirmed diagnosis per the syphilis diagnosis criteria (10). Two laboratory methods, serological-based diagnosis (treponemal and non-treponemal testing) and pathogen detection (morphology under a microscope or by polymerase chain reaction), are recommended for laboratory diagnosis in China. All the diagnosed syphilis cases were classified as primary, secondary, tertiary, congenital or latent infections. Data for primary syphilis and secondary syphilis outcomes were categorized as “early syphilis” infection in this study.

### Syphilis-related knowledge

An anonymous cross-sectional study assessed syphilis-related knowledge among the sentinel survey or community participants enrolled 95 counties/districts in Jiangsu province in 2015 and 2020, respectively. We defined areas as urban or rural based on the Provincial Bureau of Statistics announcement in 2020. Two questionnaire surveys created by the National Center for STD Control were administered to technical staff (Supplementary Table S1), the general population and high-risk sub-populations (Supplementary Table S2).

Overall, the study included data from 586 hospitals (294 hospitals in 2015 vs. 292 hospitals in 2020), which accounted for about 83.8% of syphilis case reports (84.0% of syphilis case reports in 2015 vs. 83.6% of syphilis case reports in 2020) in Jiangsu province. Technical staff were recruited, including the Center for Disease Control and Prevention (CDC), the Maternal and Child Health Hospital, and three hospitals in each county/district that reported the largest syphilis cases reported in 2015 and 2020.Nine staff members from each institution, consisting of syphilis prevention and control staff (*n* = 3), syphilis diagnosis and treatment physicians (*n* = 3), and laboratory testers (*n* = 3), completed the self-administered online survey. A score of 85% or higher was defined as having syphilis-related knowledge and awareness.

The survey among the general and high-risk sub-populations in each city’s urban and rural areas included questions on social-demographic variables (gender, age, ethnicity, education, home address) and syphilis prevention knowledge. The sample size for each sub-population was 300 urban residents aged 15–49, 300 rural residents aged 15–49, 400 migrants aged >18, 400 FSWs aged >18, and 400 MSM aged >18 in each city. Answering six or more questions out of eight correctly was defined as having syphilis prevention knowledge.

### Standard treatment for syphilis

Per the syphilis diagnosis and treatment guidelines, benzathine penicillin G (BPG) was considered the standard treatment for syphilis. The three hospitals with the highest number of reported syphilis cases for 2015 and 2020 in each county/district were included in this study. We reviewed 30 syphilis prescriptions randomly in each hospital and calculated the proportion of prescriptions for BPG by county/district. All prescriptions for a site were reviewed when prescriptions were less than 30.

### Ethical statement

All eligible data from CRIMS were de-identified before retrieval, and sentinel surveillance participants provided written informed consent. This study’s process and contents were approved by the institutional review board of the National Center for AIDS/STD Control and Prevention, Chinese Center for Disease Control and Prevention (No. of IRB Application ID: X140121318).

### Statistics

We assess the rate of syphilis screening by the average annual growth rate (AAGR). Temporal trends showing the different stages of syphilis incidence among the general population, syphilis prevalence, and condom use among the high-risk and concerned sub-populations were estimated using the Joinpoint regression program version 4.9.1.0, with annual percentage change (APC) and average annual percentage change (APPC) (11, 12). All results are presented in Supplementary Tables S3–S5. A thematic map of syphilis incidence trends divided into five levels by quartiles in 2010 was generated using the ArcGIS software version 10.2.2 (ESRI, Redlands, CA, United States), and color shades were used to indicate changes in incidence (the darker the color, the higher the incidence) (13). Statistical analysis included descriptive summaries and chi-square tests to compare the trends between different subgroups. All statistical tests were two-sided; a *p*-value less than 0.05 was considered statistically significant. All analyses were performed using IBM SPSS STATISTICS (version 19.0, SPSS Inc., Chicago, IL, United States).

## Results

### The number of syphilis screening

**Figure 4:**
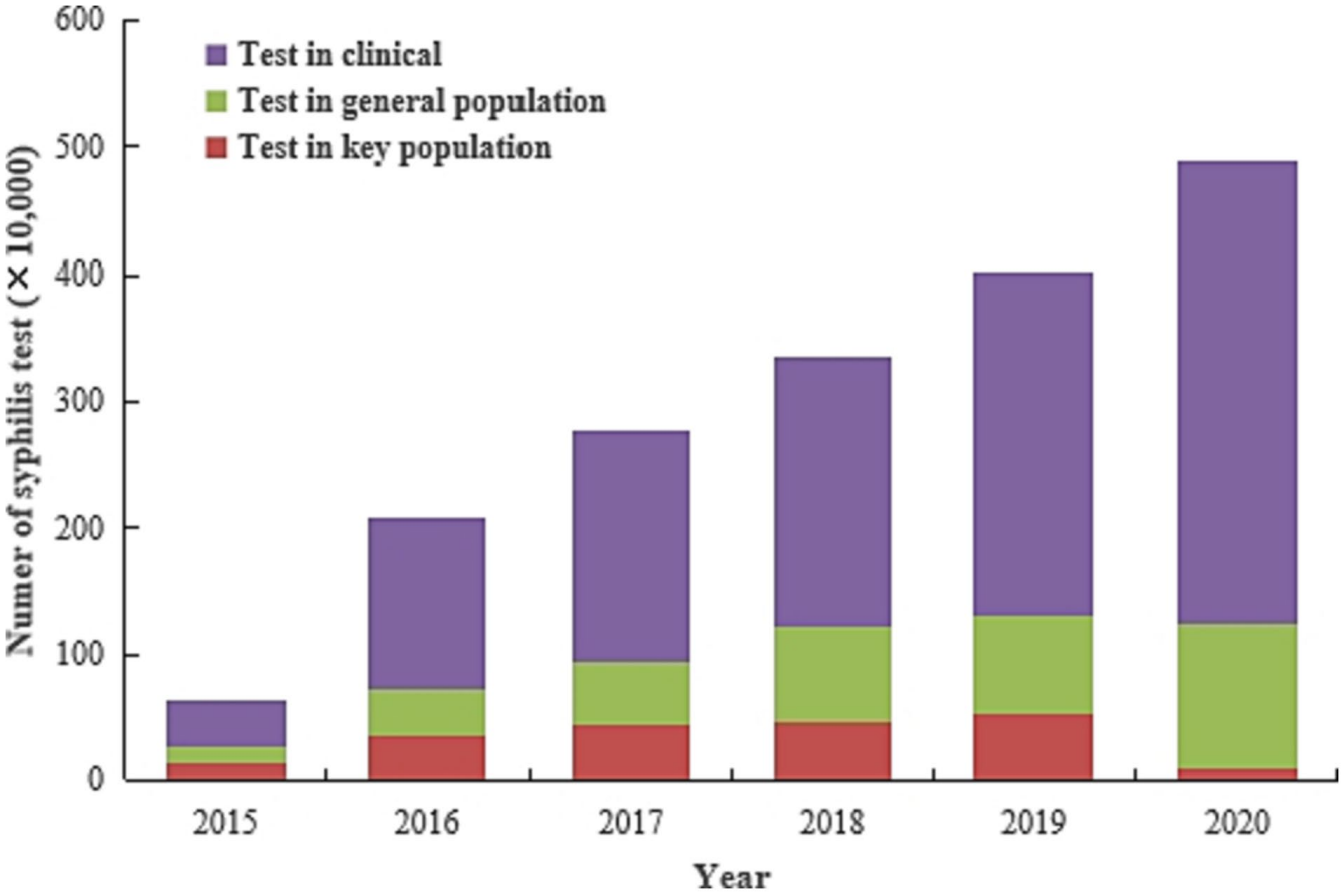
Number of syphilis screenings among various sub-groups from 2015 to 2020 in Jiangsu province, China (Data was extracted from the National Sexual Transmitted Disease Prevention and Control Management Information System (NSTDPCMIS)).

Of the 35,600,613 syphilis screenings records included in the study, 74.3% (26,437,390) between 2015 and 2020 were from clinical settings. Syphilis screenings increased from 632,887 to 4,891,219 (AAGR = 50.5%) from 2015 to 2020. The AAGR was 57.9 and 56.6% for the clinical and general populations from 2015 to 2020, respectively. Screenings among key populations increased from 130,493 to 533,785 (AAGR = 30.9%) from 2015 to 2019 but decreased to 90,275 by 2020 (Figure 4).

### Syphilis incidence trends

**Figure 5:**
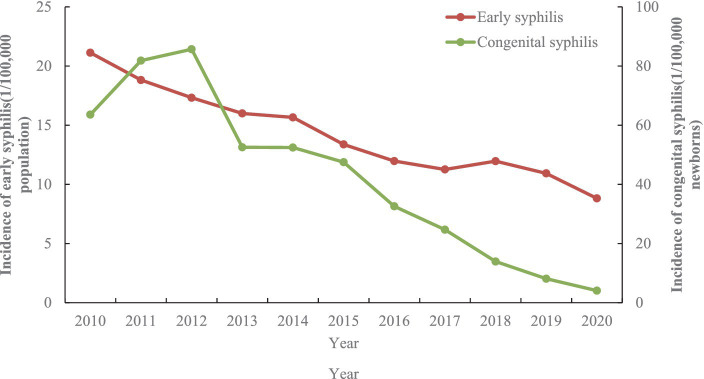
The dynamics of early and congenital syphilis incidence by year between 2010 and 2020 in Jiangsu province, China [Data was extracted from Comprehensive Response Information Management System (CRIMS)].

From 2010 to 2020 (Figure 5), the early syphilis incidence rate decreased from 21.1 to 8.8 (APC: −7.5, 95%CI: −8.6, −6.5, *p* < 0.001) per 100,000 people, and congenital syphilis incidence decreased from 63.6 to 4.1 per 100,000 newborns (APC: −14.8, 95%CI: −20.8, −8.4, *p* < 0.01).

### Spatiotemporal variations of syphilis

**Figure 6:**
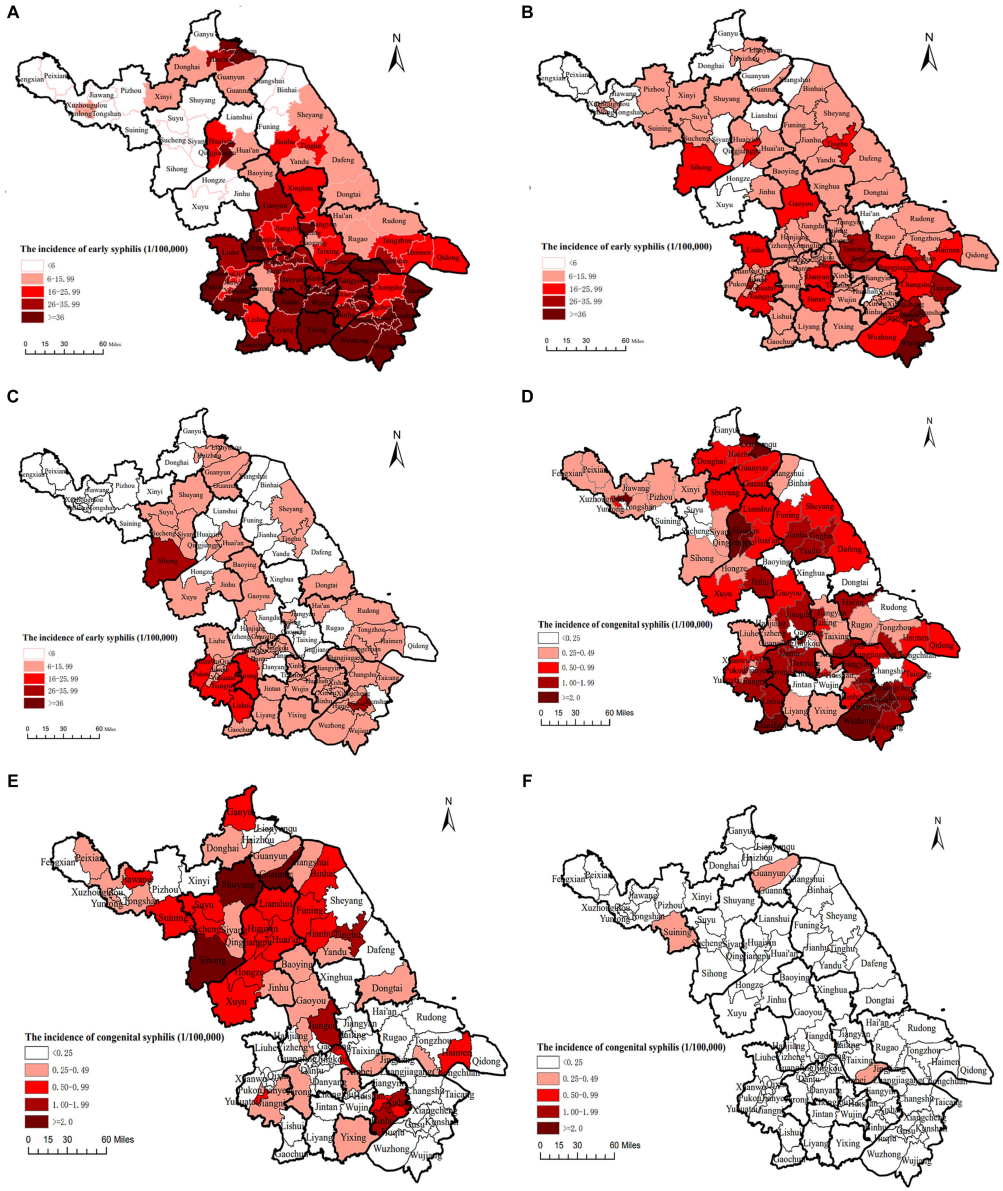
The incidence of early and congenital syphilis by geographic distribution from 2010 to 2020 in Jiangsu province, China [Date was extracted from Comprehensive Response Information Management System (CRIMS)]. **(A)** The distribution of early syphilis incidence in Jiangsu, China, 2010. **(B)** The distribution of early syphilis incidence in Jiangsu, China, 2015. **(C)** The distribution of early syphilis incidence in Jiangsu, China, 2020. **(D)** The distribution of congenital syphilis incidence in Jiangsu, China, 2010. **(E)** The distribution of congenital syphilis incidence in Jiangsu, China, 2015. **(F)** The distribution of congenital syphilis incidence in Jiangsu, China, 2020.

Figure 6 shows the spatial distribution of early and congenital syphilis in Jiangsu from 2010 to 2020. The geographic distribution of early syphilis incidence decreased significantly in all regions of the province, with higher rates in the southern regions of Jiangsu province, especially in Suzhou, Nantong, and Wuxi. Between 2010 and 2020, the incidence rate ranged from 1.3 to 185.0 per 100,000 people at the county level, and incidence rate higher than 26/100,000 decreased from 38/95 (40.0%) in 2010 to 2/95 (2.1%) in 2020 (Figures 6A–C).

Similarly, congenital syphilis was higher in the southern than in the northern regions in 2010. By 2015, the burden of congenital syphilis in the southern regions decreased. 86(90.5%) out of 95 counties reached the required target of <15/100,000 newborns by 2020 (Figures 6D–F).

### Trends in syphilis prevalence and condom use coverage among high-risk/concern sub-populations

**Figure 7:**
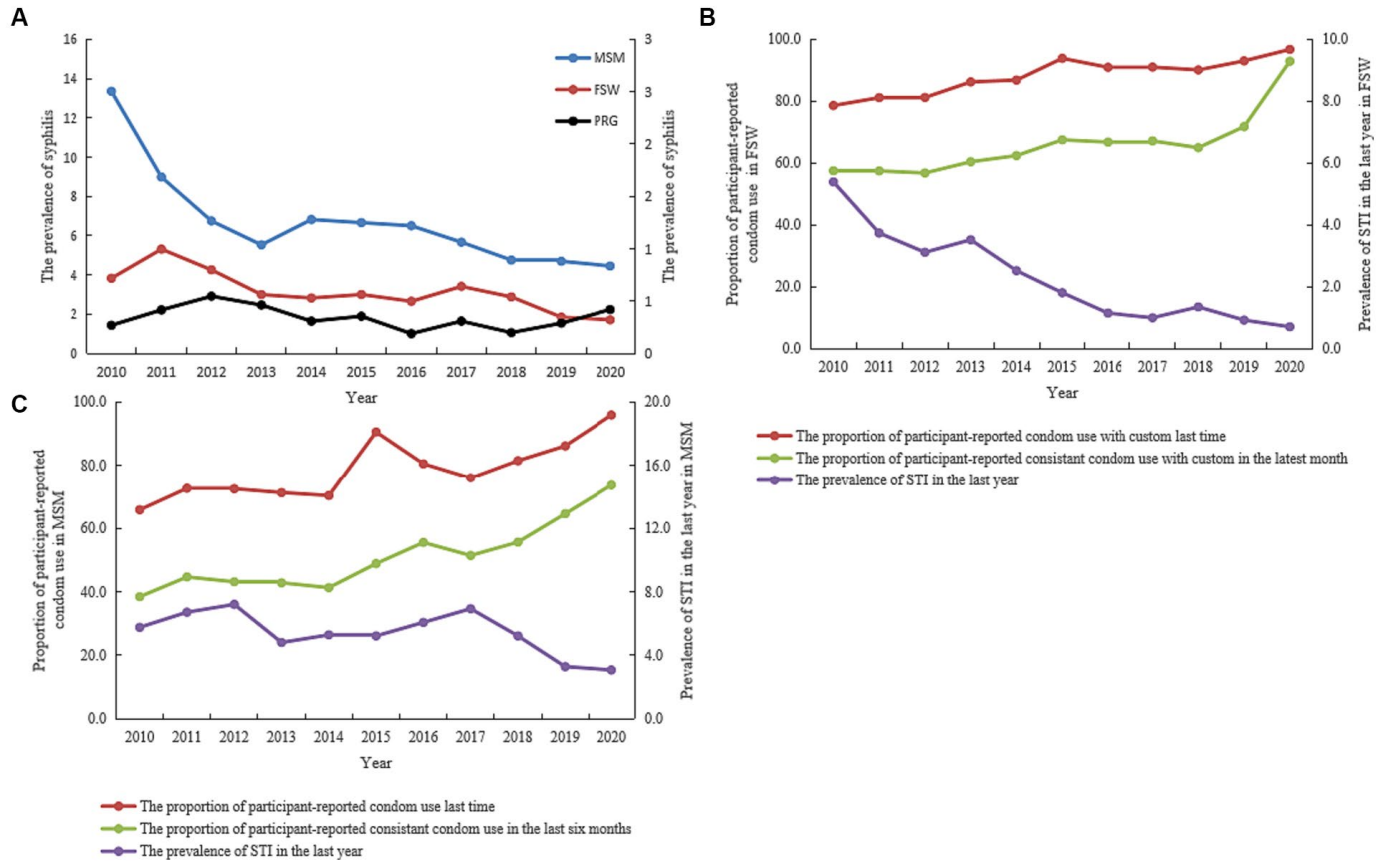
Trends showing syphilis prevalence and consistent condom use rate among high-risk/high-concerned sub-populations in Jiangsu province, China, from 2010 to 2020 (Data was extracted from the system of national STI sentinel survey). **(A)** Syphilis prevalence among female sex workers (FSW)/men who have sex with men (MSM)/pregnant women (PRG) between 2010 and 2020. **(B)** STI Prevalence and consistent condom use rate among FSW between 2010–2020. **(C)** STI prevalence and consistent condom use rate among MSM between 2010 and 2020.

Finding from the STI sentinel surveys showed that syphilis prevalence decreased from 13.4 to 3.8% (APC: −8.7, 95%CI: −12.1, −5.0, *p* = 0.001) among MSM and from 5.3 to 1.7% (APC: −7.9, 95%CI: −11.7, −3.8, *p* = 0.002) among FSW in Jiangsu from 2010 to 2020. However, prevalence remained under 1.0% and only varied slightly among PRG (APC: 0.3, 95%CI: −4.3, 5.1, *p* = 0.877) for the duration (Figure 7A).

For FSW, the reported consistent condom use rate with customers at the latest commercial sex increased from 78.6% in 2010 to 96.8% (APC: 3.2, 95%CI: 1.2, 5.1, *p* = 0.007) in 2020, and during commercial sex in the last month increased from 57.5% in 2010 to 92.9% (APC: 1.9, 95%CI: 0.7, 3.0, *p* = 0.007) in 2020. STI prevalence in the last year decreased from 5.4% in 2010 to 0.7% (APC: −17.8, 95%CI: −21.0, −14.6, *p* < 0.001) in 2020 (Figure 7B).

Similarly, reported consistent condom use at the last sexual encounter increased from 65.8 to 95.9% (APC: 3.2, 95%CI: 1.5, 4.8, *p* = 0.002), and for the last 6 months increased 38.4–73.6% (APC: 4.4, 95%CI: 1.3, 7.7, *p* = 0.013) among MSM in Jiangsu from 2010 to 2020. STI prevalence in the last year also decreased from 5.8% in 2010 to 3.1% (APC: −0.6, 95%CI: −7.7, 7.0, *p* = 0.846) in 2020 (Figure 7C).

### Syphilis-related prevention and control knowledge

**Figure 8:**
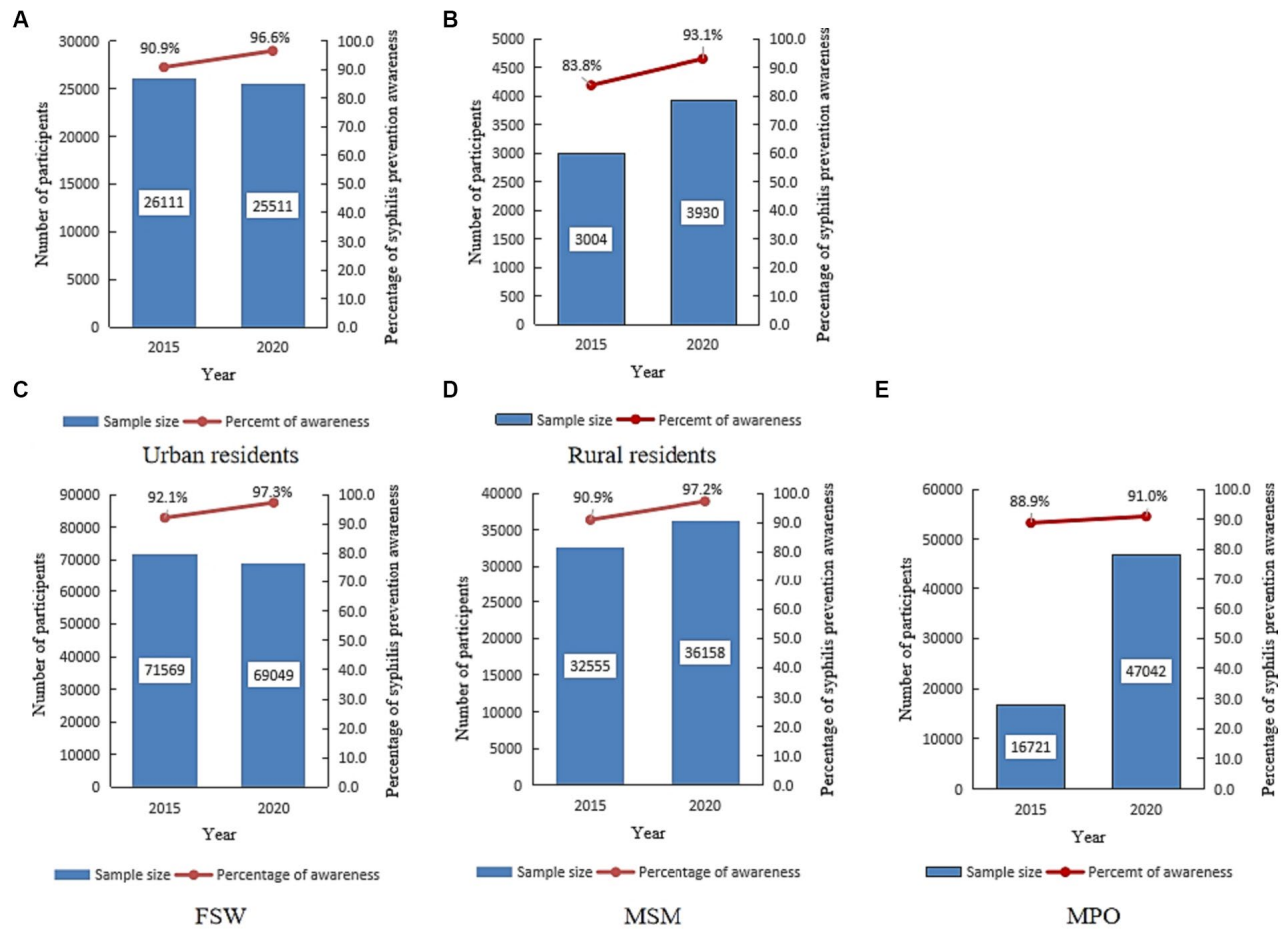
The proportion of syphilis control and prevention knowledge among various sub-populations in Jiangsu, China, in 2015 and 2020 (Data was extracted from an anonymous cross-sectional survey conducted in 2015 and 2020). **(A)** Number of participants and proportion of syphilis control and prevention knowledge among urban residents. **(B)** Number of participants and proportion of syphilis control and prevention knowledge among rural residents. **(C)** Number of participants and proportion of syphilis control and prevention knowledge among female sex workers (FSW). **(D)** Number of participants and proportion of syphilis control and prevention knowledge among men who have sex with men (MSM). **(E)** Number of participants and proportion of syphilis control and prevention knowledge among the migrant population (MPO).

Among the 58,556 general population participants, 51,622 (88.2%) were urban residents. The syphilis control and prevention knowledge coverage increased from 90.9% in 2015 to 96.6% in 2020 (Chi = 22.58, *p* < 0.001) in urban areas, and from 83.8% in 2015 to 93.1% (Chi = 36.09, *p* < 0.001) in rural areas (Figures 8A,B).

Similarly, the proportion of syphilis control and prevention knowledge increased from 92.1% in 2015 to 97.3% in 2020 (Chi = 1644.95, *p* < 0.001) among the 140,618 FSW participants and from 90.9% in 2015 to 97.2% in 2020 (Chi = 1122.64, *p* < 0.001) among the 68,713 MSM participants. For the 63,763 migrant population (MPO) participants, the proportion of syphilis control and prevention knowledge increased from 88.9% in 2015 to 91.0% in 2020 (Chi = 53.73, *p* < 0.001) (Figures 8C–E).

Among the 7,039 professional staff participants, 3,781(95.4%) passed the examination in 2015, and 3,073 (99.9%) passed the examination in 2020. Syphilis control and prevention awareness among the professional staff was categorized into three subgroups by institutional affiliation (i.e., clinical institutions, CDC, and maternity and child care systems). The results are shown in Supplementary Figures S1A–C.

### Syphilis prevention in the PMTCT

**Figure 9:**
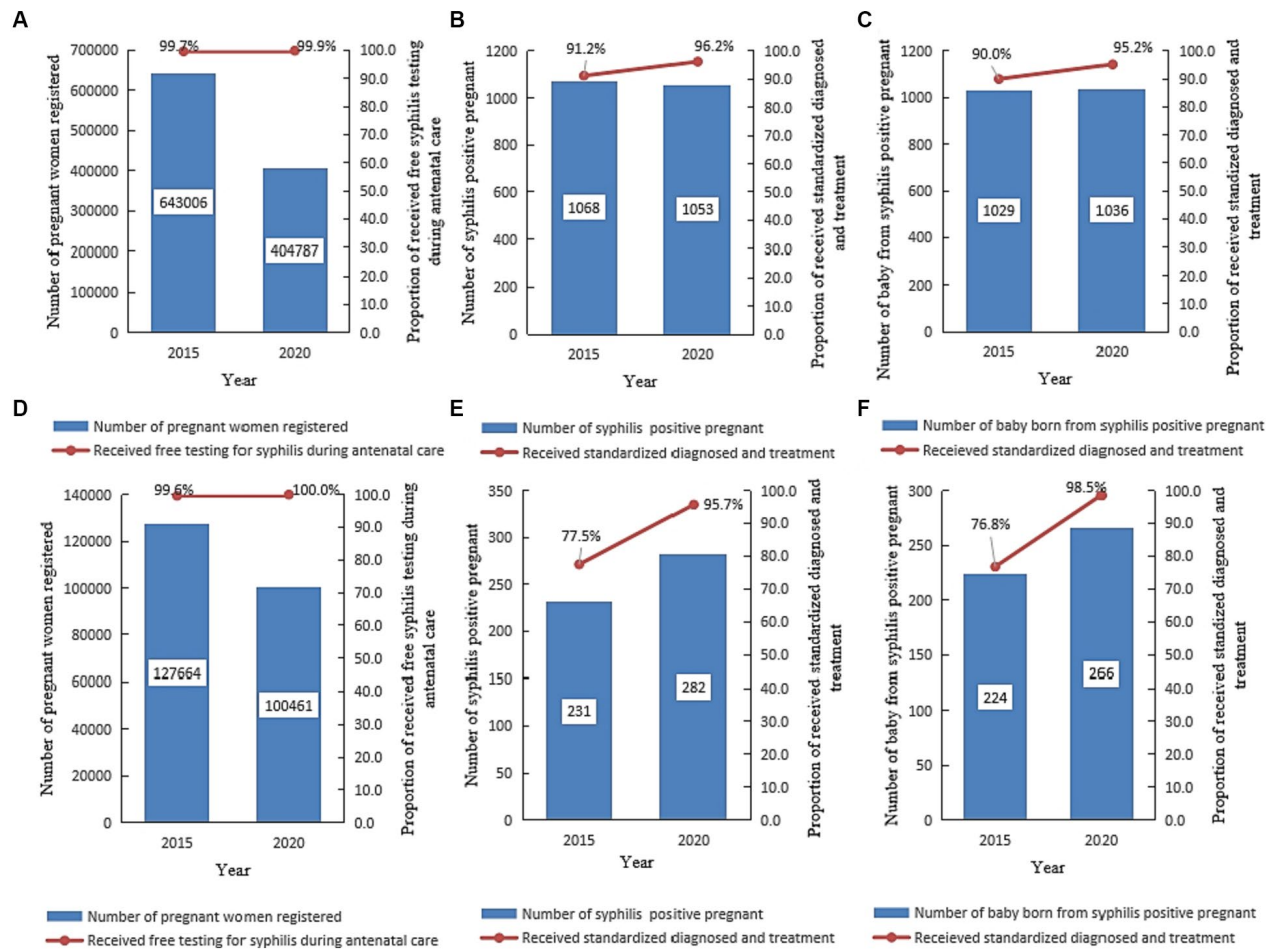
Prevention of mother-to-child transmission of syphilis in Jiangsu, China, in 2015 and 2020 [Date was extracted from the prevention of the mother-to-child transmission of syphilis, HIV, and hepatitis B (PMTCT) program]. **(A–C)** Shows the number of pregnant women registered for PMTCT, the number of pregnant women who tested positive for syphilis, and the proportion of women who received free syphilis testing, standard diagnosis, and syphilis treatment in urbanized areas in Jiangsu. **(D–F)** Shows the number of pregnant women registered for PMTCT, the number of pregnant women who tested positive for syphilis, the number of babies delivered by syphilis-positive pregnant women, and the proportion of women who received free syphilis testing, standard diagnosis, and syphilis treatment in rural areas in Jiangsu.

In urbanized areas, 1,047,793 pregnant women were registered for PMTCT. Out of this, 99.8% (10,451,157/1,047,793) received free syphilis tests during pregnancy, 2,121 pregnant women were diagnosed with current syphilis infection, and 93.7% (1,987/2,121) of them received standard diagnosis and treatment. The overall proportion receiving free syphilis testing during pregnancy (Figure 9A) increased from 99.7% (640,750) in 2015 to 99.9% (404,407) in 2020 (Chi = 653.69, *p* < 0.001). Among living births, 2,065 newborns delivered to women diagnosed with syphilis, 92.6% (1,912/2,065) of live babies received standard syphilis diagnosis and treatment services. Overall, proportion of standard syphilis diagnosis and treatment increased from 91.2% (974) in 2015 to 96.2% (1,012) in 2020 (Chi = 22.42, *p* < 0.001) among pregnant women (Figure 9B), and from 90.0% (926) in 2015 to 95.2% (986) in 2020 (Chi = 20.22, *p* < 0.001) among babies born to syphilis-positive women (Figure 9C).

In rural areas, 228,125 pregnant women were registered for PMTCT. Out of this, 99.8% (227,653/228,125) received free syphilis testing during pregnancy, 513 were diagnosed with current syphilis infection, and 87.5% (449/513) received standard syphilis diagnosis and treatment. Overall, syphilis testing during pregnancy increased from 99.6% (127,195) in 2015 to 100.0% (100,458) in 2020 (Chi = 361.53, *p* < 0.001) (Figure 9D). Of the 513 newborns delivered to syphilis-positive mothers, 95.5% (490/513) were living birth, and 45.7% (224) were born in 2015. The proportion of standard syphilis diagnosis and treatment increased from 77.5% (179) in 2015 to 95.7% (270) in 2020 (Chi = 38.76, *p* < 0.001) among pregnant women (Figure 9E), and from 76.8% (172) in 2015 and 98.5% (262) in 2020 (Chi = 56.62, *p* < 0.001) among babies born to syphilis-positive women (Figure 9F).

### Coverage of standard syphilis treatment services

**Figure 10:**
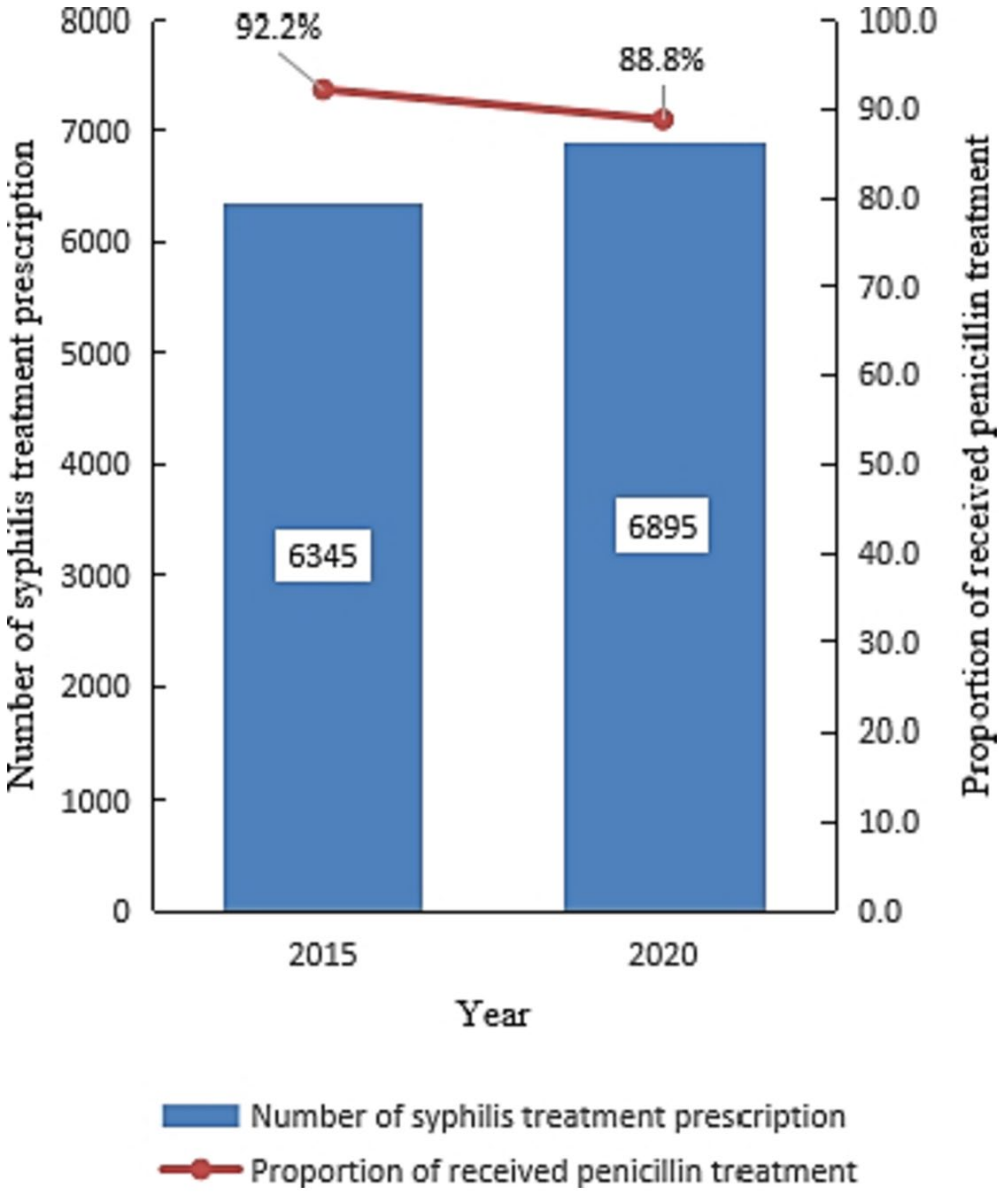
The percentage of participants who received standard syphilis treatment in 2015 and 2020 in Jiangsu, China (Date was extracted from an anonymous cross-sectional survey).

Of 13,240 prescriptions for syphilis treatment prescriptions reviewed, 47.9% (6,345) were from 2015, and 90.4% (11,974/13,240) of patients received penicillin treatment. Compared with 2015, the rate of patients who received penicillin treatment decreased in 2020 (Chi = 43.67, *p* < 0.001) (Figure 10).

## Discussion

Our study findings showed that the NSCP program contributed great progress to syphilis control, and enabled Jiangsu province to reach the 2020 milestones. Syphilis screenings increased significantly from 1,811,896 to 8,361,940, and syphilis prevention and control knowledge remained high during the period NSCP. The incidence of early syphilis and congenital syphilis among the Jiangsu population decreased from 2010 to 2020. Syphilis prevalence also decreased significantly among high-risk population (from 13.4 to 3.8% among MSM and 5.3 to 1.7% among FSW) from 2010 to 2020. The overall syphilis prevalence among pregnant women was low at 1.0%, and most (90.4%) diagnosed patients received penicillin treatment.

Since most syphilis infections are asymptomatic, there was an urgent need to maximize early diagnosis among syphilis-infected individuals by expanding coverage of syphilis screening program. Additionally, expanding access to treatment services was vital to reducing syphilis transmission routes and controlling the epidemic. The Chinese government has established several testing sites and times to facilitate syphilis screening as part of the NSCP policy. The potential entry points for syphilis screening include social settings (like entertainment centers, prisons, detention centers, DU, CSW, in-patient, surgery and STI clinics), and the established time nodes include marriage, migration, and pregnancy (14). Meanwhile, provider-initiated HIV and syphilis testing and counseling (PITC) was also provided in STD clinics in Jiangsu. The syphilis testing rate increased from 0.8% before initiating this policy to 34.3% after initiated this policy in 2015 in STD clinics. With these policies, syphilis screenings in clinical sites increased almost 10 folds, from 371,349 in 2015 to 3,644,289 in 2020.

China’s national syphilis control policy recommends that individuals with multiple sex partners, high-risk sexual behaviors, and sexual partners with a history of STI should take syphilis and HIV testing regularly. Innovative approaches such as rapid dual HIV/syphilis point-of-care diagnostic tests (POCTs) provided by community-based organizations (CBO) can help improve the syphilis screening coverage at “non-traditional” sites, especially where most traditional syphilis sites have limited time for conducting multiple tests. This is evidenced in a 2014 study where trained CBO volunteers in three Jiangsu province cities tested 512 previously untested MSM within 6 months using rapid tests. Notably, a higher proportions of MSM who tested positive received confirmatory test and linked to care (15). When CBO conducted preventive intervention and follow-up for MSM in another previous study, the HIV testing coverage among MSM increased from 4.1% in 2008 to 22.7% in 2012 in Jiangsu (16). Other decentralized testing programs also helped complement testing coverage of traditional facility-based testing. For example, an outreach team consisting of a physician, a nurse and a volunteer from CBO provided voluntary counseling and testing for HIV and syphilis in some sex venues for high-risk populations in China (17–19). With these comprehensive policies, syphilis screenings among key population almost quadrupled by 2019 (from 138,881 in 2015 to 533,785 in 2019).

The government established more than one standard STI clinic at the county level to maintain efficient treatment. Almost 1,000 healthcare institutions in Jiangsu can now provide standard syphilis treatment (treatment by BPG), following the national guidelines for syphilis diagnosis and treatment. Before the implementation of NSCP, the coverage of BPG treatment for syphilis was only 33.3% (20). By the end of 2020, the coverage of penicillin treatment for syphilis had reached 88.8% in Jiangsu. However, the coverage of BPG treatment for syphilis did not reach the final target (≥90% of the syphilis seropositive population received standard diagnosis and treatment). Several reasons might be attributed to coverage of BPG. First, the shortage of BPG still presents a major challenge in the treatment of syphilis in Jiangsu. 17% of county-level hospitals reported shortage of BPG in 2018 in Jiangsu. Second, considering the high rate of allergy to BPG, doctor probably preferred alternative drugs to BPG (21).

The integration of the syphilis care cascade, which included screening, treatment and infant follow-up, which provided universal and equitable ANC services, including syphilis and HIV diagnosis and treatment, into the PMTCT program was successful (22). With the integrated policy, maternal syphilis screenings in Jiangsu province increased from 56,009 in 2015 to 334,657 in 2020 since all pregnant women received free syphilis testing by serological methods at ANC visit. The same increasing trend was also observed in Shanghai (23). In Jiangsu province, antenatal syphilis testing coverage increased from 78.9% in 2015 to 99.9% in 2020 (24). Furthermore, the coverage of syphilis treatment for pregnant women also increased from 88.8% in 2015 to 96.1% in 2020 in Jiangsu, which was higher than the national rate (increased from 48.0 to 84.3% from 2011 to 2018) (25). However, syphilis treatment services coverage differed between urbanized and rural areas. In 2015, standard syphilis diagnosis and treatment coverage was lower for pregnant women (77.5% in rural areas vs. 91.2% in urban areas) and newborns. In rural areas, some pregnant women did not receive prenatal care due to their lack of knowledge and awareness about syphilis (26), less willingness for health care (27), and concern about leaking personal information (28). Comprehensive strategies and measures were implemented in rural areas as the NSCP implementation progress. However, this did not cause significant difference in the coverage of standard syphilis diagnosis and treatment for pregnant women and newborns between urban and rural areas by 2020.

The national sentinel surveillance system was established using serial annual cross-sectional surveys to record behavioral surveillance and laboratory testing of HIV, syphilis and HCV since 1995. For now, there were 68 sentinel sites in Jiangsu province (including 13 for MSM,28 for FSW, 6 for DUS, and 21 for CSMC), which enrolled nearly 30,000 participants for monitoring factors like their sexual behaviors, history of STIs, condom use rate, and syphilis prevalence among the different sub-groups of the resident population. The professional staff at the sentinel sites also provided counseling and referrals for all participants and free condoms for high-risk populations. Per the latest national sentinel surveillance data, four sub-populations (i.e., FSW, MSM, STD, and DU) were considered the most at-risk subgroups for HIV and other STI transmission. Integration of the NSCP program contributed to a significant decrease in the prevalence of syphilis among high-risk populations, with syphilis prevalence decreasing from 13.4 to 3.8% among MSM and from 3.8 to 1.7% among FSW from 2010 to 2020. Similar decreasing trends in syphilis prevalence were also observed among various populations in multiple provinces in China (29–32). Known factors contributing to STI prevalence among high-risk populations include inconsistent condom use (33), low STI-related knowledge (27, 28), and having multiple and casual sex partners (34). Findings of a previous population-based study in China suggested that commercial sex is more important than casual sex in the onward transmission of STIs (35). Our research indicated a significant increase in consistent condom use among both FSW and MSM from 57.5 to 92.9% and 38.4 to 73.6%, respectively, from 2010 to 2020. The increase can be attributed to NSCP’s syphilis and HIV prevention program and increased knowledge related to syphilis prevention among high-risk populations.

This study has some limitations. First, people in the early stages of a latent syphilis infection, which is contagious but asymptomatic, may be missed by clinic-based strategies for diagnosis and treatment (36). Even though people go to the hospital for some symptoms, some physicians might overlook the need to offer syphilis testing since the syphilis clinical manifestations imitate the symptoms of other diseases (37, 38). Additionally, primary or secondary syphilis infection may be misclassified as latent syphilis (39). Second, using the data from the surveillance system may have biased our estimation of the overall prevalence of syphilis due to the non-probability sampling. Additionally, our purposive inclusion of institutions engaged in evaluating the progression of the NSCP may have contributed to selection bias. Finally, syphilis cases reporting may be delayed, or patients may become lost to follow-up for treatment after diagnosis due to several reasons, including the need for outside treatment referrals, high costs to receive non-standard treatment and so on (40). Thus, our report syphilis treatment coverage among diagnosed patients could be an overestimation. However, this project was a 10-year longitudinal study with a large sample size. Our observed trends of reduction in primary and secondary syphilis incidence and syphilis characteristics (prevalence of syphilis decreased among MSM and FSW) closely mirrored findings reported by other studies conducted in the same period (41–43).

## Conclusion

Implementing the “national syphilis control plan” program has contributed to the great progress made in controlling the syphilis epidemic in Jiangsu province. The incidence of early syphilis and congenital syphilis decreased significantly from 21.1 to 8.8 per 100,000 people and from 63.6 to 4.1 per 100,000 newborns, respectively, from 2010 to 2020. The comprehensive measures, which included improving the coverage of syphilis knowledge and screening among all populations and establishing linkage to care and treatment services, were effective and should be further improved to cope with the ongoing threats to successful STI control.

## Data availability statement

The original contributions presented in the study are included in the article/Supplementary material, further inquiries can be directed to the corresponding authors.

## Ethics statement

The studies involving humans were approved by the Institutional Review Board of the National Center for AIDS/STD Control and Prevention, Chinese Center for Disease Control and Prevention. The studies were conducted in accordance with the local legislation and institutional requirements. The participants provided their written informed consent to participate in this study.

## Author contributions

LS: Formal analysis, Validation, Writing – original draft. LC: Data curation, Formal analysis, Writing – review & editing. XL: Data curation, Formal analysis, Resources, Writing – review & editing. HH: Data curation, Resources, Writing – review & editing. YHC: Data curation, Formal analysis, Resources, Writing – review & editing. YTC: Data curation, Writing – review & editing. ZZ: Data curation, Resources, Writing – review & editing. YZ: Data curation, Writing – review & editing. JL: Data curation, Formal analysis, Writing – review & editing. DY: Conceptualization, Investigation, Writing – review & editing. WG: Conceptualization, Investigation, Writing – review & editing.
