# Supplementary material for: Evaluating the effect of the plan of national syphilis control in controlling the syphilis epidemic in Jiangsu, China 2010–2020
Source: Front Public Health. 2023 Dec 21;11:1281229. doi: 10.3389/fpubh.2023.1281229 (PMC10768032; doi:10.3389/fpubh.2023.1281229)
Supplement: Supplementary file 1 [file Data_Sheet_1.docx]

**Supplement Box.1**. National syphilis control plan (NSCP) main milestones in 2015 and 2020

| Variables | National PNSC Strategy Milestones | |
| --- | --- | --- |
|  | 2015 | 2020 |
| - Proportion of awareness of syphilis-related prevention and control knowledge who work on syphilis prevention and control | Institutions of clinical and CDC: ≥85% | Institutions of clinical and CDC: 100% |
|  | Institution of maternity and child care systems: ≥80% | Institution of maternity and child care systems:≥90% |
| - Proportion of awareness of syphilis-related prevention and control knowledge among sub-population | Urban population:≥85% | Urban population:≥90% |
|  | Rural population:≥75% | Rural population:≥80% |
|  | Migrant population: ≥80% | Migrant population: ≥85% |
|  | Female sex workers: ≥90% | Female sex workers: ≥95% |
|  | Men who have sex with men: ≥90% | Men who have sex with men: ≥95% |
| - Proportion of pregnant women received syphilis screening | Urban population: ≥80% | Urban population: ≥90% |
|  | Rural population: ≥60% | Rural population: ≥70% |
| - Proportion of syphilis seropositive pregnant women received standard diagnose and treatment | Urban population: ≥90% | Urban population:≥95% |
|  | Rural population: ≥70% | Rural population:≥80% |
| - Proportion of newborns delivered from syphilis seropositive pregnant women received standard diagnose and treatment | Urban population: ≥90% | Urban population: ≥95% |
|  | Rural population: ≥80% | Rural population: ≥85% |
| - Proportation of syphilis seropositive population received standard diagnose and treatment | ≥80% | ≥90% |
| - Increment in early syphilis incidence | < 5% | < 0% |
| - Congenital syphilis incidence (1/100,000 newborns) | ≤30/100,000 newborns | ≤15/100,000 newborns |

**Supplement table 1**. A questionnaire of syphilis prevention, diagnosis and treatment and laboratory testing

| Section 1 Syphilis Prevention and Management  1. Single choice  1.1 According to the Law of Infectious Disease Prevention and Control, which kind of regulation should be followed for syphilis case report?  A. Positive reporting regulation B. Admissibility reporting regulation  C. Diagnostic and reporting regulation D. First visit doctor’s reporting regulation  1.2 According to the Law of Infectious Disease Prevention and Control, which criteria should syphilis case report meet?   1. First diagnosed syphilis and meet the national diagnostic criteria 2. Follow up syphilis and meet the national diagnostic criteria 3. Recurrent syphilis and meet the national diagnostic criteria 4. syphilis with clinical symptoms   1.3 When patient is diagnosed with syphilis, gonorrhea and genital warts, which criteria should case report meet?   1. Only one card with syphilis is filled 2. One card with syphilis is filled, another card with others is filled 3. One card with Gonorrhea is filled, another card with others is filled 4. Each card with single disease is filled.   1.4 Syphilis case report should be reported by first visit doctor, which is right for the first visit doctor?   1. By the admissions doctor diagnosis then report 2. By the doctor first diagnosis then report 3. Reported by the consultation doctor 4. Patient have been diagnosed by other hospital, then the admissions doctor diagnosis again then reprot   1.5 When we need to modify some mistakes for the infectious disease report card, which option is right?   1. Reported card can be modified casualness 2. After the card is modified, the doctor should sign 3. Modified by the supervisor, the amendment does not need permit by responsible doctor 4. Report card cannot be modified   1.6 Which of the following is the best option for filling in " syphilis report card"?   1. Fill in with a pen 2. Report card should be filled correctly, complete, no missing items 3. Should be completed by the first doctor, and signed 4. Above all right   1.7 According to the Ministry of Health issued the diagnostic criteria, which is the right for syphilis classified?   1. Early syphilis, late syphilis 2. First syphilis, Second syphilis, tertiary syphilis, Recessive syphilis, Fetal syphilis 3. Dominant syphilis, recessive syphilis 4. Acquired syphilis, congenital syphilis   1.8 Which of the following is correct for the time limit for syphilis case reports?   1. syphilis cases should be reported within 24 hours after diagnosis 2. Syphilis cases should be reported after 3 working days 3. Syphilis cases detected positive, should be reported within 24 hours 4. Syphilis cases positive, should be reported in 3 working days   1.9 Which of the following is correct about syphilis referral cases report?   1. Reported by the original referral doctor 2. Reported by attending doctor 3. Reported by the original referral doctor and the attending doctor 4. No report   1.10 Which of the following is correct about syphilis consultation cases?   1. Consultation for the first diagnosis case, reported by the original attending doctor 2. Consultation for the first diagnosis case, reported by the consultation doctor 3. Reported by original attending doctor and consultation doctors 4. No reported   1.11 According to the Ministry of Health issued the diagnostic criteria, the criteria of syphilis diagnosis can be classified as:   1. Confirmed cases, clinical diagnosis cases 2. Confirmed cases, suspected cases 3. Clinical diagnosis cases, pathogen carriers 4. Confirmed cases, positive test   1.12 According to the Ministry of Health issued the diagnostic criteria, the criteria of primary syphilis diagnosis should include:   1. Polymorphic rash, both macroscopic rapid plasma reagin (RPR) and Treponema pallidum particle agglutination assay (TPPA) were positive 2. Hard chancre, both RPR and TPPA were positive 3. Hard chancre, RPR positive 4. Hard chancre, TPPA positive   1.13 According to the Ministry of Health issued the diagnostic criteria, the criteria of secondary syphilis diagnosis should include:   1. Polymorphic rash, both macroscopic rapid plasma reagin (RPR) and Treponema pallidum particle agglutination assay (TPPA) were positive 2. Hard chancre, both RPR and TPPA were positive 3. Hard chancre, RPR positive 4. Hard chancre, TPPA positive   1.14 According to the Ministry of Health issued the diagnostic criteria, the criteria of recessive syphilis diagnosis should include:   1. Asymptomatic, no physical signs, previously not diagnosed with syphilis infection, both RPR and TPPA were positive 2. Asymptomatic, no physical signs, had a history of syphilis, both RPR and TPPA were positive 3. Asymptomatic, no physical signs, RPR positive 4. Asymptomatic, no physical signs, TPPA positive   1.15 Two types of syphilis serum antibody (TP-ELISA and RPR) were positive for pregnant woman, who had a history of syphilis infection one year ago. Does the case need to be reported?  A. Need to report  B. No need to report  C. Report after Follow-up  D. Have no idea  1.16 Two types of syphilis serum antibody (TP-ELISA and RPR) were positive for an inpatient, who did not be diagnosis syphilis before. Does the case need to be reported?  A. Need to report  B. No need to report  C. Report after Follow-up  D. Have no idea  1.17 Two types of syphilis serum antibody (TP-ELISA and tolulized red unheated serum test (TRUST)) were positive for an inquirer in Voluntary Consultation and Testing Clinic. Does the case need to be reported?   1. Report immediately 2. Referral to sexual transmitted disease (STD) clinic, the latter determine whether to report 3. Do not report, nor referral 4. Have no idea   1.18 When the blood donors were screened syphilis seropositive, how to deal with them?   1. Not reported, nor referral 2. Reported and referral 3. Not reported, referral to STD clinic 4. Have no idea   1.19 How to deal with secondary syphilis suspected cases in the comprehensive response information management system?   1. Revise the diagnosis as soon as possible and delete the case if it can’t be revised 2. The case was removed directly on the comprehensive response information management system (CRIMS) on December 31 of this year 3. If it cannot be revised, keep the case on the CRIMS 4. Have no idea   1.20 The diagnosis of Fetal syphilis is very complicated. If the patient can’t be diagnosed immediately by the laboratory test results right now, the child should be follow-up. For the child with positive TPPA test, the end point of follow-up is:  A. 3 months  B. 6 months  C. 12 months  D. 18 months  1.21 Some people without syphilis clinical symptoms and history of syphilis, received RPR-positive, TPPA negative, the appropriate diagnosis is:  A. First syphilis  B. Second syphilis  C. Recessive syphilis  D. Not syphilis  1.22 A senior doctor diagnosis a case of recessive syphilis, which of the following is correct:   1. Reported by an intern 2. Reported by a training doctor 3. Reported by graduate students 4. Reported By himself   1.23 A patient had been diagnosed of recessive syphilis in A city (a hospital has been reported cases). Then the patient had been diagnosed of recessive syphilis in B city. But the doctor noticed that the patient had been diagnosed of recessive syphilis in A city and reported in CRIMS, which of the following is correct:   1. The second hospital does not need to report 2. The second hospital needs to report 3. The second hospital needs to Follow-up and Report 4. Have no idea   1.24 About the outpatient log of syphilis case, which of the following is the appropriate option?   1. Only registered as syphilis, not registered syphilis stage 2. Registered syphilis stage 3. Do not register 4. Have no idea   1.25 When the inpatient doctor recorded the log of syphilis patients, which of the following is a suitable option:   1. Only registered as syphilis, not registered syphilis stage 2. Registered syphilis stage 3. Do not register 4. Have no idea   1.26 A baby received TPPA positive, RPR negative in a hospital. His mother had a history of syphilis. There were not any more information about his mother. Which of the following is the appropriate option?   1. Reported fetal syphilis immediately 2. According to follow-up results to determine whether to report 3. Do not reported 4. Have no idea   1.27 A women had a history of syphilis during pregnancy. Baby received RPR-positive (titer, 1:8), TPPA positive. His mother received RPR-positive (titer, 1:1), TPPA positive when delivery. Which of the following is a suitable option:   1. Report fetal syphilis immediately 2. Follow-up 3. Do not report 4. Have no idea   1.28 A patient had a history of genital ulcers 4 month ago. Now, this patient received RPR-positive, TPPA positive in a STD clinic. Which stage should be recorded for this patient?  A. Primary syphilis  B. Secondary syphilis  C. Recessive syphilis  D. Have no idea  1.29 There were not any category information for syphilis in a healthcare information system (HIS) in a hospital. When physical doctor diagnosed secondary syphilis, which of the following is a suitable option:   1. Continue to record syphilis in the HIS 2. Record secondary syphilis in paper card, do not record in HIS 3. Do not record 4. Have no idea   1.30 A new hospital do not register in the CRIMS. When syphilis patient was diagnosed in this hospital, which of the following is a suitable option:   1. Do not report 2. Record this patient, and send the card to CDC then report 3. Misdiagnosed as eczema to avoid punish 4. Have no idea   1.31 There were not any category information for syphilis in a healthcare information system (HIS) in a hospital. The attend doctor diagnosed syphilis. When Public Health Physician record  these information in CRIMS, which of the following is a suitable option:   1. Record syphilis and select syphilis stage randomly 2. Contact with attend doctor and clarify and record syphilis stage 3. Record as primary syphilis 4. Have no idea   1.32 A local center for disease control and prevention staff found that there were 5 syphilis cases record wrong syphilis stage, how to deal with these cases?   1. Remove the case and report it again 2. Revised and recorded in detail 3. Do not need any revise 4. Have no idea   1.33 A women had been diagnosed syphilis, and did not receive timely treatment. Baby received RPR and TPPA negative. Which of the following is a suitable option for baby report:   1. Due to RPR and TPPA were negative for newborn, indicating that the newborn did not suffer from syphilis, do not report fetal syphilis, not follow-up 2. Do not report fetal syphilis, but need to follow up, and according to follow-up results to determine whether the need to report 3. Due to the mothers are not timely treatment of syphilis, newborns should be immediately reported fetal syphilis 4. Have no idea   1.34 When did the China’s national ten-year syphilis control plan, named the Plan of National Syphilis Control (PNSC), initiate?  A. 2000  B. 2005  C. 2010  D. 2015  1.35 How many types of sexually transmitted diseases except AIDS?  A. 3  B. 2  C. 5  D. 32  1.36 What is the most effective prophylactic methods?   1. Oral or injecting antibiotics before and after sex 2. Use condoms correctly during sex 3. Using soap to clean the genitals before and after sex 4. Using effective sterilization drugs after sex   1.37 Which type of sexual transmitted diseases can transmit by placenta?  A. Human papillomavirus  B. Gonorrhoeae  C. Treponema pallidum  D. Haemophilus multiflora  1.38 Which type is the primary prevention for syphilis?   1. Using condoms correctly and persistently 2. Screening syphilis in sexually transmitted diseases clinic 3. Use effective therapeutic drugs 4. Referral screening positive recipients   1.39 There are key sub-populations for syphilis screening except?  A. Female sex workers  B. VCT outpatient clinic  C. College Students  D. Sexually transmitted disease outpatients  1.40 By the end of 2015, what is the aim of incidence of primary and secondary syphilis for China’s national ten-year syphilis control plan?  A .3%  B .5%  C. 8%  D 10%  1.41 Which is the level of prophylaxic measure that encourage young people to avoid premarital sex?  A. Level 1 prevention  B. Level 2 prevention  C. Level 3 prevention  D. None of above  1.42 Which of the following behavior is the most high-risk action for sexually transmitted diseases?   1. Unprotective oral sex between men 2. Unsafe intercourse sex with female sex workers 3. Have sex with net-friends 4. Have non-inserted sex female sex worker   1.43 Which option is the important indicator of evaluating sexually transmitted disease?  A. Sexual knowledge changed  B. Rate of condom use  C. The prevalence of STD among high-risk populations  D. The awareness rate of STD prevention knowledge in various populations  2. Judgment questions  2.1 According to the Law of Infectious Disease Prevention and Control, the criteria of syphilis case reporting implements screening positive report.  2.2 According to the Law of Infectious Disease Prevention and Control, the criteria of syphilis case reporting implements attending report.  2.3 According to the Law of Infectious Disease Prevention and Control, syphilis reported cases should have clinical symptoms.  2.4 According to the Law of Infectious Disease Prevention and Control, syphilis reported cases should meet the criteria of diagnosis for syphilis.  2.5 According to the Law of Infectious Disease Prevention and Control, syphilis case report implements first diagnose report.  2.6 When a patient received syphilis and gonorrhea both, one card should be reported, and syphilis is the only report case.  2.7 When a patient received syphilis and gonorrhea both, one card report syphilis, another card report gonorrhea.  2.8 Syphilis case reported by first doctor. The first doctor refers to first diagnosis syphilis.  2.9 Syphilis case reported by first doctor. The first doctor refers to attending doctor first diagnosis syphilis.  2.10 Follow-up syphilis should be reported again.  2.11 The mistake can not be revised when doctor report syphilis.  2.12 When doctor revise the syphilis case report, he should mark and sign.  2.13 A patient can report syphilis infection only according to laboratory test result. If not report, it is a mistake.  2.14 The mistake can be revised by preventive healthcare doctor.  2.15 Mis-classification on the syphilis stage can be revised by preventive healthcare doctor.  2.16 Mis-classification on the syphilis stage only can be revised by attending doctor.  2.17 The physical doctor should co-operate preventive healthcare doctor to make sure the quality of syphilis case report.  2.18 The outpatient log only records syphilis without any information of syphilis stage, when patient is diagnosed as syphilis infection.  2.19 The outpatient log should records syphilis stage, when patient is diagnosed as syphilis infection.  2.20 The inpatient log should records syphilis stage, when patient is diagnosed as syphilis infection by resident physician.  2.21 Syphilis case can be divided into early-stage syphilis and late-stage syphilis.  2.22 Syphilis cases should be reported within 24 hours after diagnosis.  2.23 Syphilis cases should be reported within 3 days after diagnosis.  2.24 Syphilis screening positive should be reported within 24 hours.  2.25 Doctors who do not have a ability to diagnose syphilis, then suspected syphilis cases should be referral or consult other professional doctor.  2.26 Syphilis referral cases should be reported by the original referral doctor.  2.27 Syphilis referral cases should be reported by the attending doctor.  2.28 Syphilis consultation cases should be reported by the consultation doctor.  2.29 Syphilis consultation cases should be reported by the consultation doctor and first doctor.  2.30 Syphilis referral cases should be reported by the attending doctor and referral doctor.  2.31 According to the Law of Infectious Disease Prevention and Control, levels of syphilis diagnose were confirmed cases and clinical diagnose cases.  2.32 According to the Law of Infectious Disease Prevention and Control, levels of syphilis diagnose were confirmed cases and suspected cases.  2.33 According to the Law of Infectious Disease Prevention and Control, levels of syphilis diagnose were clinical diagnose cases and suspected cases.  2.34 According to the Law of Infectious Disease Prevention and Control, levels of syphilis diagnose were clinical diagnose cases and pathogenic carrier.  2.35 According to the Law of Infectious Disease Prevention and Control, levels of syphilis diagnose were confirmed cases and pathogenic carrier.  2.36 Suspected syphilis cases should be revised by follow-up.  2.37 Suspected syphilis cases that can’t be revised should delete directly.  2.38 If two types of syphilis serum antibody were positive, patient should be reported.  2.39 If inpatients received syphilis screening and got positive results, patients should be reported.  2.40 A patient received TPPA and RPR positive results. The experimenter replaces physical doctor to report syphilis case without a diagnosis.  2.41 A patient without any clinical symptom was diagnosed as primary syphilis by a cardiologist. The public health physician persuades the cardiologist to revised recessive syphilis.  2.42 A dermatologist treated a secondary syphilis in a top three level hospital in September 2012. The patient received TPPA and RPR (1:4) positive in the process of follow-up in October  2014. Since there were not any clinical symptoms, the public health physician suggested the doctor record recessive syphilis and report again.  2.43 Syphilis case with a RPR titer less than 1:8 should not be reported.  2.44 A patient, had a history of unprotective sex with female sex workers, did not recorded as syphilis infection and received TPPA and RPR screening positive results. But there was not any  clinical symptom. Attending doctor did not report syphilis in CRIMS since did not test syphilis antibody in cerebrospinal fluid.  2.45 A drug user received TP-ELISA and TRUST positive in a methadone treatment clinic, the attending doctor reported recessive syphilis in the CRIMS.  2.46 Blood donors who had syphilis screening positive should be reported recessive syphilis.  2.47 Patients, had syphilis screening positive, should be diagnosed and reported by dermatologist.  2.48 Patient have RPR or TRUST titer higher than 1:8 needs to be reported.  2.49 Hospital ranked less than secondary level should not report syphilis case.  2.50 Hospital ranked more than primary level should report syphilis case.  2.51 Private medical institutions cannot report syphilis cases.  2.52 Private medical institutions had a obligation to report syphilis cases.  2.53 Newborn without any symptom should be reported recessive syphilis.  2.54 There is no need to be reported syphilis infection if patients older than 70 years.  2.55 If patient was diagnosed as HIV and syphilis infection, we only need record and report HIV infection.  2.56 If more than 2 years Child was diagnosed syphilis infection by Mother-to-child transmission, we should report as tertiary syphilis.  2.57 If somebody, without any clinical symptom and history of syphilis treatment, received TPPA and RPR positive results, these people should be reported recessive syphilis.  2.58 Blood donor received syphilis screening positive result, should be referral to STD clinic.  2.59 Patient was diagnosed as secondary syphilis two years ago. He received TPPA and RPR (1:2) positive in the latest follow up. The attending doctor reported as recessive syphilis considering  more than two years history of syphilis infection.  2.60 There are more than 20 types of sexually transmitted disease.  2.61 Sexually transmitted disease can transmit by polluted daily necessities except sex contact.  2.62 Syphilis counseling and testing services should be provided by voluntary prenatal medical examination.  2.63 Sexually transmitted disease can transmit by reproductive tract.  2.64 Daily contact, such as handshaking, eating with syphilis patients can not transmit syphilis.  2.65 Patients who got sexually infectious disease are more easy to got HIV.  2.66 If people get sexually transmitted disease, they will not get infection again because of immunity.  2.67 Latex condoms must not use lubricants for lubricants.  2.68 Not all lesions that occur in the genital area are sexually transmitted diseases.  2.69 According to the national ten-year syphilis control plan, the prevalence of congenital syphilis should less than 35/100,000 newborns at the end of 2015.  Section 2 Syphilis diagnose and treatment  1. Single choice  1.1 How many years did we divide syphilis stage of early and late in China?  A. 1 year  B. 2 years  C. 3 years  D. 4 years  1.2 Early syphilis include many stage of syphilis except?  A. Primary syphilis  B. Secondary syphilis  C. Early latent syphilis  D. Tertiary syphilis  1.3 About syphilis, which of the following statement is wrong:  A. Syphilis is a chronic and systemic sexually transmitted disease  B. Late syphilis have more contagious  C. Patients with recessive syphilis did not have any syphilis-related clinical symptoms  D. Late syphilis is more destructive to the tissues  1.4 About the biological characteristics of Treponema pallidum, which of the following is wrong:  A. Small and slender spiral microbe  B. Refractive stronger than other spirochetes  C. Can move regularly  D. Can be observed with gram stains in an optical microscope  1.5 About the biological characteristics of Treponema pallidum, which of the following is wrong:  A. Zoonosis  B. Cannot be cultured in vivo  C. The most suitable survival temperature is 37 ℃  D. Treponema pallidum can be killed easily by general disinfectants  1.6 About syphilis, which of the following is wrong:  A. Treponema pallidum reproduces heavily can cause diseases  B. Treponema pallidum can transmit by damaged skin membranes  C. Body can produce anticardiolipin antibodies and anti-Treponema pallidum antibodies in early stage of syphilis  D. Anti-Treponema pallidum antibody has an immunoprotective effect  1.7 The incubation period for primary syphilis is:  A 3～5 days  B 2～4 weeks  C 1～2 months  D 2～3 months  1.8 During the primary syphilis, the obvious clinical symptoms are:  A. Genital ulcers  B. Urethral secretions  C. Genital multiple blisters  D. Genital neoplasm  1.9 The typical manifestation of chancre own many characteristics, except:  A. Single ulcer  B. Mucus purulent discharge  C. Cartilage hardness  D. No conscious pain and tenderness  1.10 How long will the hard chancre last?  A 1～3 weeks  B 3～8 weeks  C 8～12 weeks  D 12～16 weeks  1.11 About the hard chancre, which of the following is wrong:  A. Occur on the site of Treponema pallidum implantation  B. Occur on the site of perianal and rectum among male and male sexes  C. Typical hard chancre is a single ulcer  D. Atypical chancre is painless  1.12 About the characteristics of lymph nodes near genitalia, which of the following is wrong:  A. Occur after hard chancre 1-2 weeks  B. Without swelling and heat pain  C. The lymph nodes contain Treponema pallidum  D. Enlarged lymph nodes have a soft texture and can cause adhesions  1.13 Which stage of syphilis does hard chancre occur?  A. Primary syphilis  B. Secondary syphilis  C. Tertiary syphilis  D. Congenital syphilis  1.14 When does secondary syphilis occur after Treponema pallidum infection?  A 1～3 weeks  B 3～7 weeks  C 7～10 weeks  D 10～14 weeks  1.15 Which stage of syphilis does flat condyloma belong to?  A. Primary syphilis  B. Secondary syphilis  C. Tertiary syphilis  D. Congenital syphilis  1.16 About skin and mucous membrane damage at secondary stage of syphilis, which of the following is wrong?  A. Rash diversity, widely and symmetrical distributed  B. No symptoms or mild symptoms  C. Destructive to the tissues  D. Highly contagious  1.17 About skin and mucous membrane damage at secondary stage of syphilis, which of the following is wrong?  A. Pimples and macula are rare  B. The skin lesions on the palms and soles are dark red or light brown circular desquamation lesions  C. Skin shows "raw ham" color  D. Mostly distributed in the trunk and limbs  1.18 There are many characteristics for flat warts, except:  A. Occur in the perianal, external genital area  B. The skin lesion is pimples or plaque  C. Clear boundaries  D. Without Treponema pallidum usually  1.19 The following is the clinical manifestations of secondary syphilis, except:  A. Superficial lymph nodes enlarge  B. Eye syphilis  C. Pleomorphic skin and mucous membrane damage  D. Gumma  1.20 The following is the clinical manifestations of secondary syphilis, except:  A. Headache, nausea and other precursor symptoms  B. Can occur moth-like hair loss  C. Neurosyphilis is mostly asymptomatic  D. Bone and joint pain increases during daytime and activity  1.21 Which stage of syphilis is gumma?  A. Primary syphilis  B. Secondary syphilis  C. Tertiary syphilis  D. Neurosyphilis  1.22 Which is the skin and mucous membrane syphilis characteristic?  A. Multiple skin lesions with symmetrical distribution  B. Rapid progress, not self-healing  C. Rarely ulcers  D. Inflammation and constitutional symptom are mild  1.23 Which stage of syphilis is paralytic dementia?  A. Primary syphilis  B. Secondary syphilis  C. Secondary recurrent syphilis  D. Neurosyphilis  1.24 Neurosyphilis can be divided into many types, except:  A. Meningeal nerve syphilis  B. Meningeal blood vessels syphilis  C. Brain parenchymal syphilis  D. Eye syphilis  1.25 Which stage of syphilis is spinal tuberculosis?  A. Primary syphilis  B. Secondary syphilis  C. Secondary recurrent syphilis  D. Neurosyphilis  1.26 Paralytic dementia has various clinical manifestations, except:  A Occurred after 10 to 20 years syphilis infection  B Mental retardation, inattention  C Arrow pupil  D Optic nerve atrophy  1.27 Spinal tuberculosis has various clinical manifestations, except:  A. Lightning pain  B. Optic nerve atrophy  C. Feeling abnormal  D. Tactile sensation and temperature disorder  1.28 About latent syphilis, which of the following is wrong:  A. Without any clinical symptoms  B. Serum antibody of syphilis is positive  C. The duration time for early latent syphilis is less than two years  D. No infectious  1.29 Early congenital syphilis has various clinical manifestations, except:  A. Nutritional disorders  B. Hard chancre  C. Blister or blister rash  D. Flat condyloma  1.30 About congenital syphilis, which of the following is true:  A. The longer pregnant women infected, the more likely to transmit to fetus  B. Can occur hard chancre  C. Rhinitis is the most common early symptoms  D. Not easy to involve the nervous system  1.31 Syphilis serum fixation means:  A. After fully treatment, Treponema pallidum antibody contain positive in a certain period of time  B. After fully treatment, non-Treponema pallidum antibody contain positive in a certain period of time  C. Untreated, the titer of non-Treponema pallidum antibody keeps at the same level in 3 consecutive tests  D. Untreated, the titer of Treponema pallidum antibody keeps at the same level in 3 consecutive tests  1.32 There are many purposes of syphilis treatment, except:  A Treponema pallidum should be eliminated at any stage of syphilis  B Damage disappeared and cured at early stage of syphilis  C Each stage of syphilis are required seroconversion  D Prevent fetus from infection for congenital syphilis  1.33 Recommended treatment plan for early syphilis is:  A. Benzathine penicillin G 1.2 million U, on both sides of the buttocks intramuscularly, once a week, 2 times  B. Benzathine penicillin G 2.4 million U, on both sides of the buttocks intramuscularly, once a week, 2 times  C. Benzathine penicillin G 4.8 million U, on both sides of the buttocks intramuscularly, once a week, 2 times  D. Benzathine penicillin G 1.2 million U, on both sides of the buttocks intramuscularly, once a week, 5 to 6 times  1.34 The preferred treatment for early syphilis is:  A. Penicillin sodium  B. Penicillin potassium  C. Ceftriaxone  D. Benzathine penicillin  1.35 When patient had a history of allergy for Penicillin, the preferred treatment for early syphilis:  A Doxycycline 100mg, 2 times a day, oral, 15 days  B Azithromycin 500mg, 1 time a day, oral, 15 days  C Erythromycin 500mg, 4 times a day, oral, 15 days  D Doxycycline 200mg, 2 times a day, oral, 15 days  1.36 The preferred treatment for late stage of syphilis is:  A. Benzathine penicillin G 1.2 million U, on both sides of the buttocks intramuscularly, once a week, 3 times  B. Benzathine penicillin G 1.2 million U, on both sides of the buttocks intramuscularly, once a week, 6 times  C. Benzathine penicillin G 4.8 million U, on both sides of the buttocks intramuscularly, once a week, 3 times  D. Benzathine penicillin G 240 million U, on both sides of the buttocks intramuscularly, once a week, 3 times  1.37 When patient had a history of allergy for Penicillin, the preferred treatment for late stage of syphilis is:  A Doxycycline 100mg, 2 times a day, oral, 30 days  B Azithromycin 250mg, 2 times a day, oral, 30 days  C Azithromycin 500mg, 1 time a day, oral, 30 days  D Erythromycin 500mg, 4 times a day, oral, 30 days  1.38 Water penicillin treatment for neurosyphilis, the dose range is:  A 4.80～6.4 million U/day  B 6.4～8 million U/day  C 10～12 million U/day  D 18～24 million U/day  1.39 The appropriate description of the treatment for early stage of syphilis is:  A. Intravenous drip benzathine penicillin  B. Intramuscular inject Benzathine penicillin  C. Intravenous drip penicillin sodium  D. Intramuscular inject Penicillin sodium  1.40 It has been confirmed that Treponema pallidum has been generally resistant to the following drugs:  A. Benzathine penicillin  B. Procaine penicillin  C. Macrolides  D. Tetracycline  1.41 According to drug metabolic half-life, which of the following is correct:  A. Water penicillin> procaine penicillin> benzathine penicillin  B. Benzathine penicillin> procaine penicillin> water agent penicillin  C. Water penicillin> benzathine penicillin> procaine penicillin  D. Benzathine penicillin> water agent penicillin> procaine penicillin  1.42 About ceftriaxone treatment early stage of syphilis, which of the following is wrong:  A. Considering its long half-life, so it can be administered once a week  B. Good penetration into cerebrospinal fluid  C. Only as an alternative treatment  D. Have treatment effect on Treponema pallidum in animal model  1.43 There are many characteristics for Kyrgyzstan reaction, except:  A. Often occurs in several hours after the first dose of treatment  B. Fever, cold, general malaise, headache, musculoskeletal pain and so on  C. Pregnant women can cause premature birth or fetal intrauterine asphyxia  D. The incidence is higher at late stage of syphilis than early stage of syphilis  1.44 If treatment is effective, how long does non-Treponema pallidum antibodies remain positive?  A. Primary syphilis last 3 months; secondary syphilis last 6 months  B. Primary syphilis last 6 months; secondary syphilis last 12 months  C. Primary syphilis last 9 months; secondary syphilis last 18 months  D. Primary syphilis last 12 months; secondary syphilis last 24 months  1.45 According to the criteria of early syphilis treatment effective, how many times does the titer of non-Treponema pallidum antibodies decline compared with baseline?  A Two times  B Four times  C Eight times  D Twelve times  1.46 How long does early syphilis follow-up last?  A 6 months  B 6～12 months  C 12～24 months  D 24～36 month  1.47 About pregnant women, which of the following is wrong for syphilis treatment?  A Treatment after clarified  B Should screen non- Treponema pallidum antibodies titer after treatment during pregnancy  C Treatment with erythromycin or doxycycline if Penicillin allergy  D Erythromycin can’t pass through the placenta, the fetal treatment is invalid  1.48 The plan of prophylactic treatment for adult is:  A Benzathine penicillin G 1.2 million U, intramuscularly, once a week, 2 times  B Benzathine penicillin G 240 million U, intramuscularly, once a week, 2 times  C Benzathine penicillin G 1.2 million U, intramuscular injection, 1 time  D Benzathine penicillin G 240 million U, intramuscular injection, 1 time  1.49 The recommend plan of early congenital syphilis with cerebrospinal fluid abnormalities is:  A Water infusion of penicillin G, 10 million U ~ 15 million U / (kg • d), within 7 days after birth, each 50,000 U / kg, intravenous injection every 12 hours 1; Infants treated 1 time every 8 hours until the total course of treatment 10 ~ 14 d  B Enamel penicillin, 100,000 U ~ 150,000 U / (kg • d), newborns within 7 days after birth, with a daily dose of 50,000 U / kg, 1 time every 12 hours; Infants treated 1 time every 8 hours until the total course of 10 ~ 14 d  C Camphor penicillin, 100,000 U ~ 150,000 U / (kg • d), newborns within 7 days after birth, every 50,000 U / kg, intravenous 1 time every 12 hours; Infants treated 1 time every 8 hours until the total course of 10 ~ 14 d.  D Erythromycin, 100,000 U ~ 150,000 U / (kg • d), newborns within 7 days after birth, every 5 000 U / kg, intravenous 1 time every 12 hours; Infants treated 1 time every 8 hours until the total course of 10 ~ 14 d  1.50 About cerebrospinal fluid for Neurosyphilis, which of the following is wrong:  A White blood cell count ≥ 5 × 106 / L  B Protein> 500mg / L  C VDRL can be replaced by RPR  D The specificity of the Treponema pallidum is more obvious  1.51 In the prenatal examination, the best period of syphilis testing for pregnant women is:  A Early pregnancy  B Mid-pregnancy  C Late pregnancy  D Time of birth  1.52 Which of the statement for pregnancy syphilis is wrong:  A The main transmission route of maternal infection with syphilis is sex  B Pregnancy syphilis often have obvious clinical symptoms  C Pregnancy syphilis easily leads to abortion, stillbirth, congenital syphilis or neonatal death and other adverse outcomes  D At any stage of pregnancy, Treponema pallidum can infect the fetus  1.53 Which of the following test results can determine pregnant women are currently infected with syphilis and are contagious:  A RPR（－） TPPA（－）  B RPR（＋） TPPA（－）  C RPR（＋） TPPA（＋）  D RPR（－） TPPA（＋）  1.54 Which of the following statement for congenital syphilis is right:  A. If mother had a history of syphilis infection, baby received RPR positive can be diagnosed as congenital syphilis  B. If mother had a history of syphilis infection, baby received TPPA positive can be diagnosed as congenital syphilis after 18 months’ follow up  C. Baby received TPPA and RPR (1:1) positive can be diagnosed as congenital syphilis after 18 months’ follow up  D. Baby did not infect syphilis if we did not detect Treponema pallidum in dark field microscope  1.55 Which situation can exclude the possibility of congenital syphilis when newborn delivered?  A Detected Treponema pallidum in dark field microscopy  B TPPA positive, RPR titer 4 times higher than mother’s  C Treponema pallidum IgM antibody positive  D TPPA positive, RPR negative  1.56 The following description of the prophylactic treatment of children born by pregnant women with syphilis is wrong:  A Mothers do not receive full treatment during pregnancy, adequate treatment, children should receive prophylactic treatment  B Mothers are treated with non-penicillin, and children should receive prophylactic treatment  C Benzathine penicillin G, 50,000 units/kg body weight, sub-double gluteal muscle injection after newborn delivery  D Prophylactic treatment should last 15 days  **2. Judgment questions**  2.1 Early syphilis is the Treponema pallidum infected within 3 years.  2.2 Early syphilis includes primary syphilis, secondary syphilis and early latent syphilis.  2.3 Late syphilis includes late benign syphilis, cardiovascular syphilis and late latent syphilis.  2.4 Neurosyphilis can occur in early and late stage of syphilis.  2.5 The incubation time of the hard chancre last 4-8 weeks.  2.6 Typical hard chancre is painless or non-tenderness.  2.7 The hard chancre only occurs near the genitals or the anus.  2.8 The enlarged lymph node in the primary syphilis is painful, and the surface is red and swollen.  2.9 If the time of syphilis infection is less than 2-3 weeks, non-Treponema pallidum antibody might be negative in primary syphilis.  2.10 Skin lesion can imitate any skin lesion in secondary syphilis.  2.11 Condyloma latum is a typical damage in secondary syphilis.  2.12 Oral mucosa is a typical damage in tertiary syphilis.  2.13 Plaque like Alopecia is a typical damage of secondary syphilis.  2.14 Skin lesions are widely and symmetrical distribution in secondary syphilis.  2.15 Superficial lymph nodes can be enlarged in secondary syphilis.  2.16 Neurosyphilis can occur in secondary syphilis.  2.17 It is easy to find treponema pallidum in the condyloma latum or [moist papule](http://www.youdao.com/w/moist%20papule/#keyfrom=E2Ctranslation) in secondary syphilis.  2.18 Non-treponema pallidum antibody is positive in secondary syphilis.  2.19 Gumme is a typical damage in secondary syphilis.  2.20 Perforation on upper palate and septum and saddle nose are usually seen in tertiary syphilis.  2.21 Meningoencephalitis is a type of neurosyphilis.  2.22 Cerebral parenchymal syphilis includes paralytic dementia and the tuberculosis of the spinal cord.  2.23 The typical characteristics of locomotor ataxia is the concentration and personality disorder.  2.24 The count of white blood cell decreased significantly in cerebrospinal fluid during neurosyphilis.  2.25 The non-Treponemal pallidum antibody in cerebrospinal fluid can help doctor to diagnose neurosyphilis.  2.26 The Treponemal pallidum antibody in cerebrospinal fluid can help doctor to diagnose neurosyphilis.  2.27 Early congenital syphilis share similar clinical feature with adult secondary syphilis.  2.28 Late congenital syphilis share similar clinical feature with adult tertiary syphilis.  2.29 Congenital syphilis can be diagnosed by positive test of the non-Treponemal pallidum antibody.  2.30 Congenital syphilis can be diagnosed by positive test of the Treponemal pallidum IgM antibody.  2.31 If the tier of non-Treponemal pallidum antibody is 4 times higher than mother’s, the newborn can be diagnosed with congenital syphilis.  2.32 Congenital syphilis can be diagnosed by positive test of the Treponemal pallidum antibody for newborn.  2.33 If the Treponemal pallidum antibody is positive at 15 month’s follow-up for a baby, then the baby can be diagnosed with congenital syphilis.  2.34 The earlier treatment for syphilis infection received the better effect.  2.35 The irregular treatment for syphilis infection can increase recurrence and lead to advanced damage earlier.  2.36 The period of follow up lasts one year to evaluate the effect of early syphilis treatment.  2.37 The first option is Benzathine penicillin for early syphilis treatment.  2.38 The first option is Benzathine penicillin for neurosyphilis.  2.39 The first option is Aqueous penicillin for late syphilis.  2.40 The first option is Benzathine penicillin for early congenital syphilis with cerebrospinal fluid abnormal.  2.41 It has been found that many treponema spirals resistant to penicillin.  2.42 It has been found that many treponema spirals resistant to macrolide antibiotics.  2.43 The “jihai reaction” caused by non-Treponemal pallidum antibody remain positive after treatment.  2.44 Non-Treponemal pallidum antibody seroconversion occurs in 6 months in most patients.  2.45 The recurrence of syphilis is that Non-Treponemal pallidum antibody is positive again or the titer of non-Treponemal pallidum antibody increased 4 times than last test.  2.46 The period of follow up last more than 3 years for cardiovascular syphilis or neurosyphilis.  2.47 Serum fixation means non-Treponemal pallidum antibody remains positive for a long time after treatment.  2.48 When pregnant women receive maternal health care services for the first time, we should provide free syphilis screening.  2.49 The standard treatment must include two courses of treatment for pregnancy syphilis. Also, the interval time of two course should be more than two weeks. The last course of treatment  must provide penicillin, ceftriaxone or erythromycin and treat at late pregnancy.  2.50 If mother had a treatment for syphilis infection during pregnancy, the newborn did not receive prophylactic treatment.  2.51 Suspected congenital syphilis should be follow up regularly to confirm the status of syphilis infection.  2.52 Pregnant women diagnosed syphilis infection during labor should be treated one course.  2.53 If Treponema pallidum IgM antibody is negative, the newborn should not be diagnosed with congenital syphilis.  2.54 Newborn without any cerebrospinal fluid abnormal can be treated with benzathine penicillin, according to 50,000 U / kg body weight for a muscle injection.  Section 3 Syphilis Laboratory Testing  1. Single choice  1.1 Which is the appropriate staining for treponema pallidum?  A. Gram stain  B. Methylene blue staining  C. Silver stain  D. Hematoxylin-eosin staining  1.2 Which is the appropriate method for non- treponema pallidum antibody?  A. TPPA  B. ELISA  C. RPR  D. FTA-ABS  1.3 Which antibody does the Treponema pallidum particle agglutination assay (TPPA) test?  A. IgA .  B. IgG  C. IgG,IgM, IgA, et al  D. IgM  1.4 Which specimen is not available for treponema pallidum detect?  A. Tissue fluid of skin ulcer  B. Amniotic fluid  C. Blood  D. Cerebrospinal fluid  1.5 Which specimen is not available for non- treponema pallidum antibody test?  A. [Serum](http://www.youdao.com/w/serum/#keyfrom=E2Ctranslation)  B. Blood plasma  C. [Whole blood](http://www.youdao.com/w/whole%20blood/#keyfrom=E2Ctranslation)  D. Cerebrospinal fluid  1.6 Which specimen is not available for enzyme linked immunosorbent assay (ELISA)?  A. [Serum](http://www.youdao.com/w/serum/#keyfrom=E2Ctranslation)  B. Blood plasma  C. [Whole blood](http://www.youdao.com/w/whole%20blood/#keyfrom=E2Ctranslation)  D. Cerebrospinal fluid  1.7 The etiology detection method for Treponema pallidum does not include:  A. [Dark-field microscopy](http://www.youdao.com/w/dark-field%20microscopy/#keyfrom=E2Ctranslation)  B. Silver stain  C. Medium culture method  D. Nucleic acid test  1.8 What is the speed for horizontal rotator in RPR or TRUST test?  A. (100±2) rotations per minute  B. (100±10) rotations per minute  C. (120±2) rotations per minute  D. (120±10) rotations per minute  1.9 What is the diameter for horizontal rotator in RPR or TRUST test?  A. (25±2) mm  B. (18±2) mm  C. (20±2) mm  D. (30±2)mm  1.10 How long does the RPR or TRUST reaction in the horizontal rotator?  A. 5 minute  B. 8 minute  C. 10 minute  D.12 minute  1.11 How many microliters per well does TRUST test?  A. 50 μl  B. 30 μl  C. 25 μl  D.17 μl  1.12 Which of the following method does the frontal zone phenomenon occur?  A. ELISA  B. RPR  C. TPPA  D. FTA-ABS  1.13 How many microliters per well does RPR test?  A. 50 μl  B. 30 μl  C. 25 μl  D.17 μl  1.14 There are many approaches to test non-treponemal antibodies except?  A. RPR  B. ELISA  C. VDRL  D. TRUST  1.15 How many time does the dilution ratio for TPPA test?  A. 1:20  B. 1:40  C. 1:80  D. 1:160  1.16 Which type of the antigen is in ELISA?  A. Recombinant antigen  B. Complete treponema pallidum  C. Ultrasonic pyrolysis of treponema pallidum  D. lipid content  1.17 Which type of the antigen is in TPPA?  A. Recombinant antigen  B. Complete treponema pallidum  C. Ultrasonic pyrolysis of treponema pallidum  D. lipid content  1.18 Which type of the antigen is in RPR?  A. Recombinant antigen  B. Complete treponema pallidum  C. Ultrasonic pyrolysis of treponema pallidum  D. lipid content  1.19 What is the biosafety level (BL) for syphilis serological test?  A. BL-1  B. BL-2  C. BL-3  D. BL-4  1.20 What is the main purpose of external quality control?  A. The effectiveness of detecting approach  B. The accuracy of detecting result  C. The veracity of detecting result  D. The feasibility of detecting approach  1.21 What is the main aim of internal quality control?  A. The effectiveness of detecting approach  B. The accuracy of detecting result  C. The veracity of detecting result  D. The feasibility of detecting approach  1.22 Which type of antibody can be only detected by RPR?  A. IgA  B. IgG  C. IgG,IgM, IgA, et al  D. IgM  1.23 Which type of method does treponemal pallidum antibodies test?  A. TPPA  B. ELISA  C. RPR  D. VDRL  1.24 Which type of method can not be used screen syphilis for blood specimen?  A. TRUST  B. ELISA  C. PCR  D. TPPA  1.25 Which stage of syphilis can not be detected by etiology test?  A. Primary syphilis  B. Secondary syphilis  C. latent syphilis  D. Neurosyphilis  1.26 Which stage of syphilis can be diagnosed by treponemal pallidum IgM antibody?  A. Congenital syphilis  B. Secondary syphilis  C. latent syphilis  D. Pregnant syphilis  1.27 Which stage of syphilis can be diagnosed by cerebrospinal fluid VDRL test?  A. Primary syphilis  B. Secondary syphilis  C. latent syphilis  D. Neurosyphilis  1.28 Which type of method is the most valuable method for neurosyphilis diagnosis?  A. Blood TRUST  B. Blood ELISA  C. Blood RPR  D. [Cerebrospinal fluid](http://www.youdao.com/w/cerebrospinal%20fluid%20(CSF)/#keyfrom=E2Ctranslation) VDRL  1.29 Which type of method of syphilis detecting need microscope?  A. TRUST  B. ELISA  C. RPR  D. VDRL  1.30 Which type of method of syphilis detecting can be use whole blood sample?  A. TRUST  B. ELISA  C. RPR  D. Immunochromatography rapid detection  1.31 Which type of method is high sensitivity for detecting serum antibody after syphilis infection?  A. TRUST  B. TPPA  C. RPR  D. VDRL  1.32 Which type of antibody can’t be transmitted by placenta when mother is diagnosed syphilis infection?  A. IgM  B. IgG  C. All of the antibodies  D. IgA  1.33 What is the main object for treponemal pallidum detection in dim view microscope?  A. Treponemal pallidum of typical movement  B. Typical dye treponemal pallidum  C. Typical inflammatory cells  D. Typical pathology changes  1.34 What is the main object for treponemal pallidum detection in silver-plated staining?  A. Treponemal pallidum of typical movement  B. Typical dye treponemal pallidum  C. Typical inflammatory cells  D. Typical pathology changes  1.35 How many does the odds of non-treponemal pallidum in secondary syphilis?  A. <90%  B. 100%  C. <95%  D. >95%  1.36 How many does the odds of treponemal pallidum in secondary syphilis?  A. <90%  B. 100%  C. <95%  D. >95%  1.37 Which of the following quantitative test method is useless for evaluating therapeutic effect?  A. TRUST  B. TPPA  C. RPR  D. VDRL  1.38 How long does the maximum follow up time last for congenital syphilis diagnosis by treponemal pallidum antibody?  A. Six month  B. Nine month  C. Twelve month  D. Eighteen month  1.39 If frontal zone phenomenon is suspected in RPR or TRUST test, how many times the serum should be diluted at least?  A. 1:2  B. 1:4  C. 1:8  D. 1:16  1.40 Which type of method should be adopted to mix antigen and antibody in TPPA test?  A. Horizontal gyro  B. Hand blender  C. 96-well-plate oscillator  D. Suspended implement  1.41 Which type of method should be adopted to mix antigen and antibody in RPR test?  A. Horizontal gyro  B. Hand blender  C. 96-well-plate oscillator  D. Suspended implement  1.42 What is the reason if treponemal pallidum antibody test by RPR/TRUST?  A. There are typical non-treponemal pallidum antibodies  B. There aren’t typical non-treponemal pallidum antibodies  C. There are typical treponemal pallidum antibodies  D. There are non-treponemal pallidum and treponemal pallidum antibodies both  1.43 When one sample was quantified by RPA and TRUST, which of the following opinion is right about the titer?  A. Not necessarily same  B. Definitely same  C. The titer of RPR must be higher than TRUST  D. The titer of TRUST must be higher than RPR  1.44 What is the golden standard method for treponemal pallidum antibodies testing?  A. ELISA  B. TPPA  C. Chemiluminescent immunoassay  D. Immunochromatography rapid detection  1.45 Which type of specimen is not appropriate for treponema pallidum detection by PCR test?  A. [Interstitial fluid](http://www.youdao.com/w/interstitial%20fluid/#keyfrom=E2Ctranslation)  B. Lymph fluid  C. Cerebrospinal fluid  D. Blood  1.46 Which kind of method is appropriate for syphilis screening in big sample size?  A. ELISA  B. TPPA  C. RPR/ TRUST  D. Immunochromatography rapid detection  1.47 Which type of method can be used to exclude biological false positive, when RPR is positive in syphilis screening?  A. TRUST  B. TPPA  C. VDRL  D. RPR  1.48 Which type of antibody can not pass through the blood-brain barrier?  A. IgM  B. IgG  C. All of the antibodies  D. IgA  1.49 Which stage of syphilis can be diagnosed by treponema pallidum IgM antibody?  A. Primary syphilis  B. Secondary syphilis  C. latent syphilis  D. Neurosyphilis  1.50 According to the pathogenicity, which level is the treponema pallidum?  A. First kind  B. Second kind  C. Third kind  D. Fourth kind  2. Judgment questions  2.1 If somebody is diagnosed as syphilis infection, treponema pallidum antibody remain positive lifelong except some very early syphilis.  2.2 RPR or TRUST only can be used for syphilis screening,  2.3 Both TPPA and RPR test were 100% positive in secondary syphilis.  2.4 When someone is diagnosed as syphilis infection, then the tier of RPR should be above 1:8.  2.5 Congenital syphilis can use cord blood to test treponema pallidum antibody.  2.6 If treponema pallidum is negative by PCR in blood specimen, we can neglect the possibility of syphilis infection.  2.7 When newborn received full course treatment for syphilis, baby received TPPA positive or RPR negative can be diagnosed as syphilis infection after 18 months’ follow up.  2.8 Manual shaking can be used in PRP or TRUST quantitative test.  2.9 If subjects received TPPA negative and RPR positive, we can neglect the possibility of syphilis infection.  2.10 Cerebrospinal fluid VDRL test results must be positive if patient with neurosyphilis.  2.11 If agglutination test is not obvious in RPR test, we can prolong the reading time to 10 minutes.  2.12 There is no need to dilute serum to re-test if RPR or TRUST is negative in original sample.  2.13 If we did not detect any treponema pallidum in dark field examination in specimen of genitals, we can neglect the possibility of syphilis infection.  2.14 We need to dilute the serum to re-test if frontal zone phenomenon occurred.  2.15 Newborn can be diagnosed as congenital syphilis if he receives TPPA and RPR positive.  2.16 We can use PCR to enhance sensitivity for syphilis test, because there was a window period for syphilis serological test.  2.17 The rotation speed is (120±2) rotation per minute for RPR/TRUST test.  2.18 Someone can be diagnosed as syphilis infection if pathogen of treponema pallidum is positive.  2.19 Any kind of serological detection method can be used to syphilis screening.  2.20 People can be diagnosed as syphilis infection if TPPA was positive.  2.21 People can be diagnosed as syphilis infection if treponema pallidum IgM antibody was positive.  2.22 Since RPR and TRUST have the same principle, the results of must be consistent in quantitative experiment.  2.23 When people received TPPA positive and RPR negative, there might be window period for non-treponema pallidum antibodies. Patient should be follow up.  2.24 ELISA and other specific treponema pallidum antibody tests can be used to detect the patients who had a syphilis history.  2.25 Sero-resistance means that TPPA remain positive even though patients received standardized treatment of syphilis.  2.26 The purpose of internal quality control is to improve the precision of results.  2.27 Titer of TPPA is an important indicator for treatment efficacy.  2.28 If TPPA test is positive and RPR test was negative, it means that RPR test was false negative.  2.29 Treponema pallidum IgM antibody test can replace TPPA or other tests.  2.30 There is no need to follow standard operating procedure, as long as there is instruction for syphilis kit.  2.31 If ELISA is positive, there must be treponema pallidum antibodies in the specimen.  2.32 Histological fluid specimens can be used to detect pathogen of treponema pallidum in primary and secondary syphilis.  2.33 Whole blood sample can not be sued in TPPA or ELISA.  2.34 The agglutination can be observed by microscope, if RPR or TRUST is suspicious.  2.35 Internal quality control products can not product by same laboratory.  2.36 There is no need to check periodically for horizontal rotary instrument.  2.37 We can only test IgG antibodies using TPPA or RPR.  2.38 Treponema pallidum IgM antibodies can not be used to diagnose neurosyphilis.  2.39 Syphilis serology test should be performed in first level biosafety laboratory.  2.40 Whole blood specimen is not appropriate for immunochromatography rapid detection.  2.41 Silver plating can not be used in detection of pathogen of treponema pallidum.  2.42 We only need to follow one kind of standard operating procedure, as long as RPR and TRUST share the same principle.  2.43 Good result of external quality control means high precision of results in this laboratory.  2.44 TPPA applied Gelatin particles as vector is better than TPHA applied red blood as vector.  2.45 All syphilis serological tests should be recorded timely.  2.46 There is no need to record the methods in all syphilis serological tests.  2.47 The specimen and antigen should be mixed by the oscillator when TPPA was conducted.  2.48 The frontal zone can be eliminated by repeated qualitative test in RPR or TRUST test.  2.49 Treponema pallidum antibodies are prior to non-treponema pallidum antibodies.  2.50 If following up 18 months for newborn, congenital syphilis can be diagnosed precisely. |
| --- |

**Supplement table 2**. Questionnaire about Syphilis Prevention Knowledge for General population

| Address: ___________City, _____________County (District)  People type: ①Urban ②Rural ③Rural-to-urban migrants ④Female sexual worker ⑤Men who have sex with men  Number of questionnaire：口口口口 GB code：口口口口口口  ──────────────────────────────────  Dear, an investigation that people's awareness of some health problems is going to improve our work. This investigation does not need to sign, and we will keep your answer confidential. I hope your answer is real knowledge, and I can provide health advice for you after the investigation. Thank you!  Syphilis prevention knowledge problem:  1. Is sexual contact the main transmission route for syphilis?  ①Yes ②No ③Do not know  2. Can syphilis be cured?  ①Yes ②No ③Do not know  3. Might a person who looks healthy be a syphilis patient?  ①Yes ②No ③Do not know  4. Can condom prevent syphilis?  ①Yes ②No ③Do not know  5. Does syphilis infection increase risk of acquire HIV?  ①Yes ②No ③Do not know  6. Do sexual partners of syphilis patients need to go to a hospital for syphilis testing?  ①Yes ②No ③Do not know  7. Can pregnant women that infected with syphilis transmitted it to their fetuses?  ①Yes ②No ③Do not know  8. Can social contacting, such as eating, handshaking, with syphilis patients spread syphilis?  ①Yes ②No ③Do not know  Investigator sign： Leader sign：  Supervisor sign： Time： |
| --- |

**Supplement table 3**. Trends in incidence of early syphilis and congenital syphilis in Jiangsu, China: Year of change of trend, annual percentage change, and annual average percentage change

| Variables | Period | Join-point | APC^a^,95%CI^c^ | *P* value | AAPC^b^,95%CI^c^ | *P* value |
| --- | --- | --- | --- | --- | --- | --- |
| Early syphilis | 2010-2020 | 0 | -7.5, (-8.6, -6.5) | <0.001 | -7.5, (-8.6, -6.5) | <0.001 |
| Congenital syphilis | 2010-2020 | 0 | -19.4, (-24.0, -14.5) | <0.001 | -19.4, (-24.0, -14.5) | <0.001 |

^a^ APC, annual percentage change; ^b^ APPC, average annual percent change; ^c^ CI, confidence interval.

**Supplement table 4**. Trends in current syphilis prevalence among subgroups in Jiangsu, China: Year of change of trend, annual percentage change, and annual average percentage change

| Variables | Period | Join-point | APC^a^,95%CI^c^ | *P* value | AAPC^b^,95%CI^c^ | *P* value |
| --- | --- | --- | --- | --- | --- | --- |
| MSM | 2010-2020 | 0 | -8.7, (-12.1, -5) | 0.001 | -8.7, (-12.1, -5) | 0.001 |
| FSW | 2010-2020 | 0 | -7.9, (-11.7, -3.8) | 0.002 | -7.9, (-11.7, -3.8) | 0.002 |
| PRG | 2010-2020 | 0 | 0.3, (-4.3, 5.1) | 0.877 | 0.3, (-4.3, 5.1) | 0.877 |

^a^ APC, annual percentage change; ^b^ APPC, average annual percent change; ^c^ CI, confidence interval; MSM, men who have sex with men; FSW, female sex worker; PRG, pregnant women.

**Supplement table 5**. Trends in rate of condom using among subgroups in Jiangsu, China: Year of change of trend, annual percentage change, and annual average percentage change

| Category | Variables | Period | Join-point | APC^a^,95%CI^c^ | *P* value | AAPC^b^,95%CI^c^ | *P* value |
| --- | --- | --- | --- | --- | --- | --- | --- |
| FSW | Condom use for latest time commercial sex | 2010-2020 | 0 | 3.2, (1.2, 5.1) | 0.007 | 2.0, (1.2, 2.9) | <0.001 |
|  | Condom use for last month commercial sex | 2010-2020 | 0 | 1.9, (0.7, 3.0) | 0.007 | 4.8, (3.8, 6.1) | <0.001 |
|  | STIs prevalence in the last year | 2010-2020 | 0 | -17.8, (-21.0, -14.6) | <0.001 | -17.8, (-21.0, -14.6) | <0.001 |
| MSM | Condom use for latest time commercial sex | 2010-2020 | 0 | 3.2, (1.5, 4.8) | 0.002 | 3.2, (1.5, 4.8) | 0.002 |
|  | Condom use for last month commercial sex | 2010-2020 | 0 | 4.4, (1.3, 7.7) | 0.013 | 6.6, (3.5, 9.8) | <0.001 |
|  | STIs prevalence in the last year | 2010-2020 | 0 | -0.6, (-7.7, 7.0) | 0.846 | -7.1, (-15, 1.5) | 0.104 |

^a^ APC, annual percentage change; ^b^ APPC, average annual percent change; ^c^ CI, confidence interval; MSM, men who have sex with men; FSW, female sex worker; STIs, sexually transmitted infections.


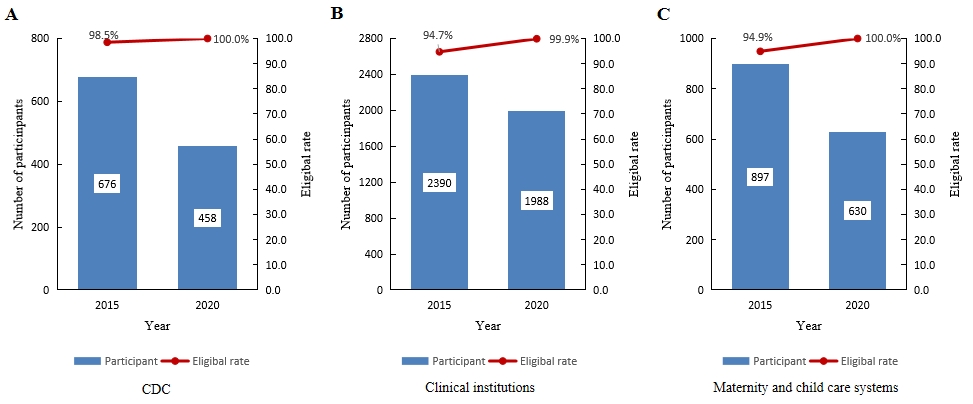


**Supplement Fig. 1**. The proportion of syphilis control and prevention knowledge among professional staff in Jiangu, China, in 2015 and 2020 (Data was extracted from an anonymous cross-sectional survey conducted in 2015 and 2020). **A.** Number of participants and proportion of syphilis control and prevention knowledge among professional staff from Center for disease control and prevention (CDC). **B.** Number of participants and proportion of syphilis control and prevention knowledge among professional staffs from clinical institutions. **C.** Number of participants and proportion of syphilis control and prevention knowledge among professional staffs in maternity and child health care systems.
